# Structural basis of archaeal FttA-dependent transcription termination

**DOI:** 10.1038/s41586-024-07979-9

**Published:** 2024-09-25

**Authors:** Linlin You, Chengyuan Wang, Vadim Molodtsov, Konstantin Kuznedelov, Xinyi Miao, Breanna R. Wenck, Paul Ulisse, Travis J. Sanders, Craig J. Marshall, Emre Firlar, Jason T. Kaelber, Thomas J. Santangelo, Richard H. Ebright

**Affiliations:** 1 Waksman Institute and Department of Chemistry and Chemical Biology, Rutgers University, Piscataway, NJ, USA.; 2 Center for Microbes, Development, and Health, Shanghai Institute of Immunity and Infection, Chinese Academy of Sciences, Shanghai, China.; 3 Department of Biochemistry and Molecular Biology, Colorado State University, Fort Collins, CO, USA.; 4 Rutgers CryoEM and Nanoimaging Facility and Institute for Quantitative Biomedicine, Rutgers University, Piscataway, NJ, USA.; 5 Present address: Research Institute of Molecular and Cellular Medicine RUDN, Moscow, Russia.; 6 These authors contributed equally: Linlin You, Chengyuan Wang, Vadim Molodtsov.

## Abstract

The ribonuclease FttA (also known as aCPSF and aCPSF1) mediates factor-dependent transcription termination in archaea^1-3^. Here we report the structure of a *Thermococcus kodakarensis* transcription pre-termination complex comprising FttA, Spt4, Spt5 and a transcription elongation complex (TEC). The structure shows that FttA interacts with the TEC in a manner that enables RNA to proceed directly from the TEC RNA-exit channel to the FttA catalytic centre and that enables endonucleolytic cleavage of RNA by FttA, followed by 5′→3′ exonucleolytic cleavage of RNA by FttA and concomitant 5′→3′ translocation of FttA on RNA, to apply mechanical force to the TEC and trigger termination. The structure further reveals that Spt5 bridges FttA and the TEC, explaining how Spt5 stimulates FttA-dependent termination. The results reveal functional analogy between bacterial and archaeal factor-dependent termination, functional homology between archaeal and eukaryotic factor-dependent termination, and fundamental mechanistic similarities in factor-dependent termination in bacteria, archaea, and eukaryotes.

FttA mediates factor-dependent transcription termination in archaea^1-3^, and is responsible for transcription termination at the majority of transcription units in archaea in vivo^3^. FttA exhibits endoribonucleolytic activity and 5′→3′ exoribonucleolytic activity^1-7^. FttA has a bi-metal nucleolytic catalytic centre, comprising two Zn^2+^ ions coordinated by six histidine residues, located in an active-centre groove formed by the interface between a metallo-β-lactamase domain (MβL) and a fused β-CASPase domain^4-7^ (β-CASP). FttA binds specifically to U-rich RNA^3^ and, as a result, performs endoribonucleolytic and 5′→3′ exoribonucleolytic activity preferentially on U-rich RNA^2,3,7^. During FttA-dependent transcription termination, FttA is thought to perform endoribonucleolytic cleavage of the nascent RNA at a position about 15 to 25 nucleotides (nt) from the 3′ end of the nascent RNA, followed by 5′→3′ exoribonucleolytic digestion of the 3′ RNA-cleavage product^1^ (Fig. 1a,b). Single-nucleotide-resolution mapping of the FttA-dependent endoribonucleolytic cleavage site using TECs assembled on synthetic nucleic acid scaffolds indicates that FttA-dependent endoribonucleolytic cleavage primarily occurs exactly 22 nt from the 3′ end of the nascent RNA (Fig. 1c).

FttA is a homologue of the RNA-cleavage subunits of the multisubunit, megadalton complexes that mediate factor-dependent transcription termination in eukaryotes^1-16^ (Fig. 1d). Thus, FttA is a homologue of INTS11, the RNA-cleavage subunit of the eukaryotic integrator complex (INT), a 14-subunit, 1.6 MDa complex involved in termination of Pol II on small nuclear RNA genes and promoter-proximally paused Pol II on mRNA genes^8-12^. FttA also is a homologue of CPSF73 (Yth1 in yeast), the RNA-cleavage subunit of the eukaryotic cleavage and polyadenylation specificity factor (CPSF (CPF in yeast)), a 21-subunit, 1.8 MDa complex involved in termination of Pol II on mRNA genes^13-16^. Archaeal FttA consists of fused MβL and β-CASP domains preceded by two K homology domains^4-7^ (KH1 and KH2) (Fig. 1d). Eukaryotic INTS11 and CPSF73 consist of fused MβL and β-CASP domains followed by C-terminal dimerization sequences^4-10,13,16^ (Fig. 1d).

NusG/Spt5 is the sole universally conserved transcription factor; it is present in bacteria, archaea, and eukaryotes^17-19^. Bacterial NusG and archaeal Spt5 each consists of a NusG N-terminal domain (NGN), which interacts with RNA polymerase (RNAP), and a Kyprides–Ouzounis–Woese domain (KOW), which interacts with factors that associate with and/or cooperate with RNAP^17-19^ (Fig. 1e). Eukaryotic Spt5 consists of an NGN domain and KOW domain followed by eukaryotic-specific C-terminal sequences^17-19^ (Fig. 1e). FttA-dependent transcription termination is stimulated by Spt5^1^ (Fig. 1c). A protein fragment corresponding to the Spt5 KOW domain is unable to stimulate FttA-dependent termination^1^, indicating that stimulation of FttA-dependent termination requires the Spt5 NGN domain or requires both the Spt5 NGN domain and Spt5 KOW domain^1^.

Bacterial factor-dependent transcription termination is stimulated by NusG, which bridges the TEC and the transcription termination factor Rho, with the NusG NGN domain interacting with the TEC, and with the NusG KOW domain interacting with Rho^20^. The fact that both bacterial Rho-dependent transcription termination and archaeal FttA-dependent transcription termination are stimulated by a member of the NusG/Spt5 protein family^1,20^ raises the possibility that bacterial Rho-dependent transcription termination and archaeal FttA-dependent transcription termination share functional homology or functional analogy. Here, to test this possibility, we used cryogenic electron microscopy (cryo-EM) to determine an atomic structure of an archaeal FttA-dependent pre-termination complex.

## Structure of the FttA pre-termination complex

**Fig. 1 ∣:**
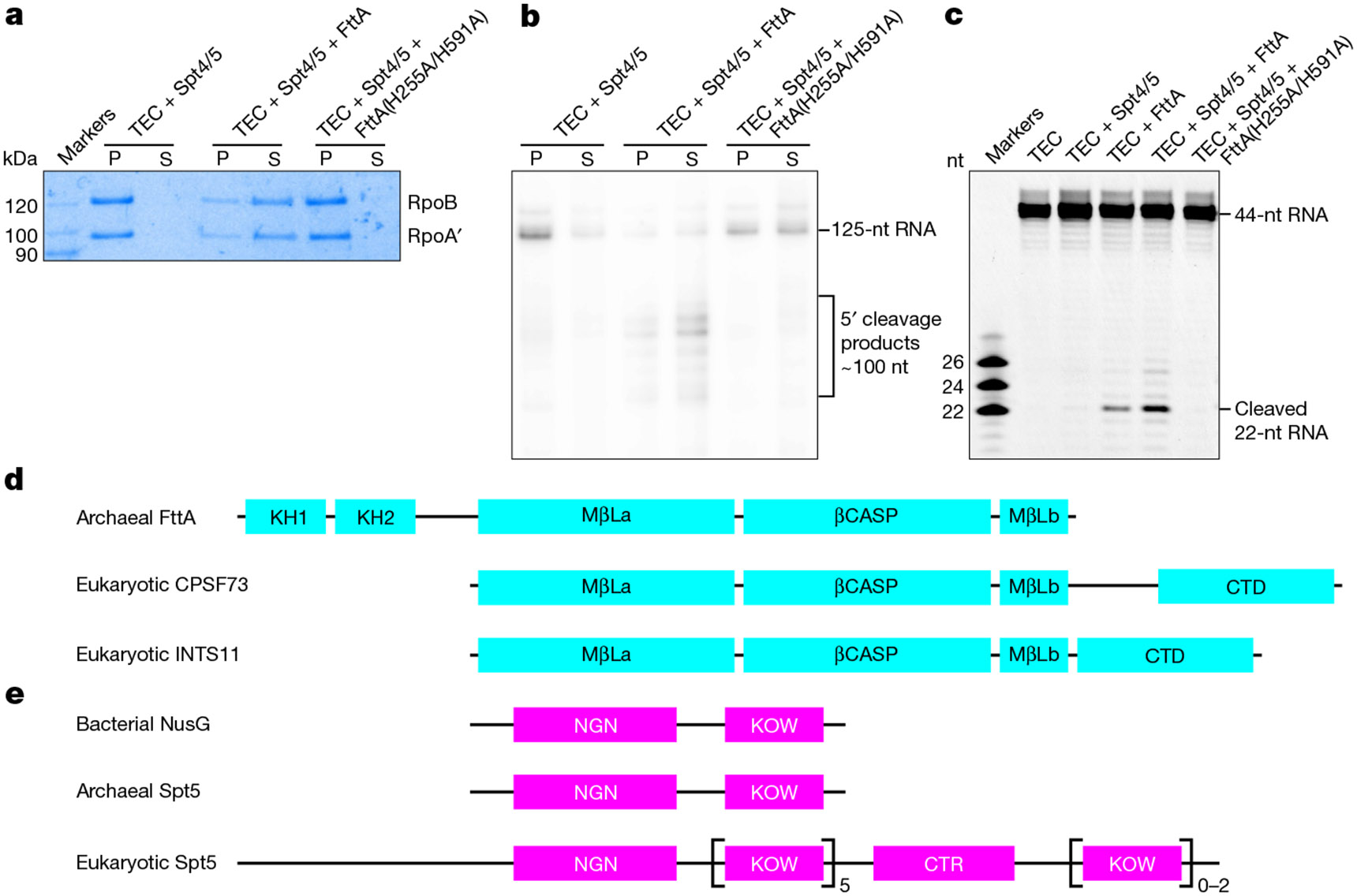
The transcription termination factor FttA and transcription elongation factor NusG/Spt5. **a**, FttA-dependent transcription termination, assessed using release assays with bead-immobilized promoter-generated TECs, detecting retained and released RNAP RpoA′ and RpoB subunits by Coomassie staining. P, pellet fraction; S, supernatant fraction; Spt4/5, Spt4–Spt5 complex. Assays were performed twice, with consistent results. **b**, FttA-dependent transcription termination and RNA cleavage, assessed using release assays with bead-immobilized promoter-generated TECs containing ^32^P-5′-end-labelled RNA, detecting retained and released RNA by storage-phosphor scanning (methods as in ref. 1). Assays were performed twice, with consistent results. **c**, Single-nucleotide-resolution mapping of FttA-dependent RNA cleavage, assessed using TECs assembled on synthetic nucleic acid scaffolds containing 44-nt fluorescein-5′-end-labelled nascent RNA, detecting intact and cleaved RNA by x/y fluorescence scanning and sizing cleavage products by comparison to fluorescein-5′-end-labelled synthetic RNAs corresponding to 22-, 24-, and 26-nt cleavage products (scaffold and marker sequences in Extended Data Fig. 1b). Assays were performed twice, with consistent results. **d**, Domain architectures of archaeal FttA, eukaryotic CPSF73, and eukaryotic INTS11^4-10,13,16^. Residue numbers for domains of *Tko* FttA are as follows: KH1, 6–75; KH2, 76–144; MβLa, 198–428; βCASP, 433–557; MβLb, 576–639. CTD, C-terminal domain. **e**, Domain architectures of bacterial NusG, archaeal Spt5, and eukaryotic Spt5^17-19^. KOW1–KOW5 are present in all eukaryotes; KOW6–KOW7 are present only in plants and metazoans. CTR, C-terminal region.

To obtain an archaeal FttA pre-termination complex, we analysed a double-mutant FttA derivative, FttA(H255A/H591A), from the hyperthermophilic archaeon *T. kodakarensis* (*Tko*). In FttA(H255A/H591A), two of the six histidine residues that coordinate Zn^2+^ ions in the FttA nucleolytic catalytic centre are substituted^4-7^. This double substitution abolishes FttA-dependent termination, FttA-dependent endonucleolytic cleavage of RNA, and FttA-dependent 5′→3′ exonucleolytic cleavage of RNA (Fig. 1a-c). Accordingly, FttA(H255A/H591A) is proficient in forming a pre-termination complex that contains both FttA and a TEC, but is deficient in subsequent steps of termination.

**Extended Data Fig. 1 ∣:**
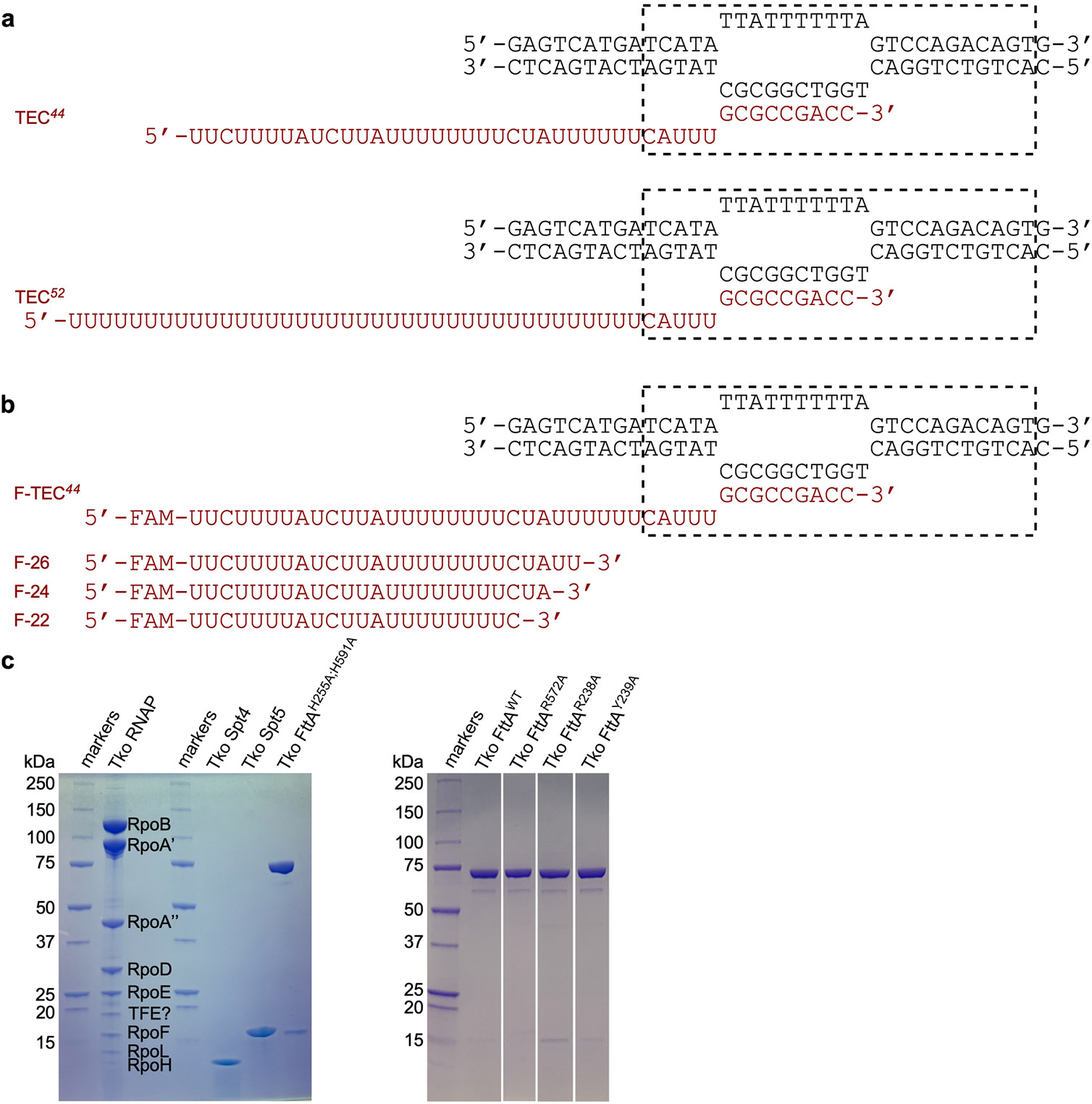
Nucleic acids and proteins. **a**, Nucleic-acid scaffolds for structural studies: TEC^44^ and TEC^52^. Black, DNA; brick red, RNA; dashed rectangle, TEC. **b**, Nucleic-acid scaffold and RNA markers for biochemical studies: F-TEC^44^, F-RNA^22^, F-RNA^24^, and F-RNA^26^. F, fluorescein. Other features as in **a**. **c**, Proteins for structural and biochemical studies (Coomassie-stained PAGE). The analysis was performed twice, with consistent results obtained.

**Extended Data Fig. 2 ∣:**
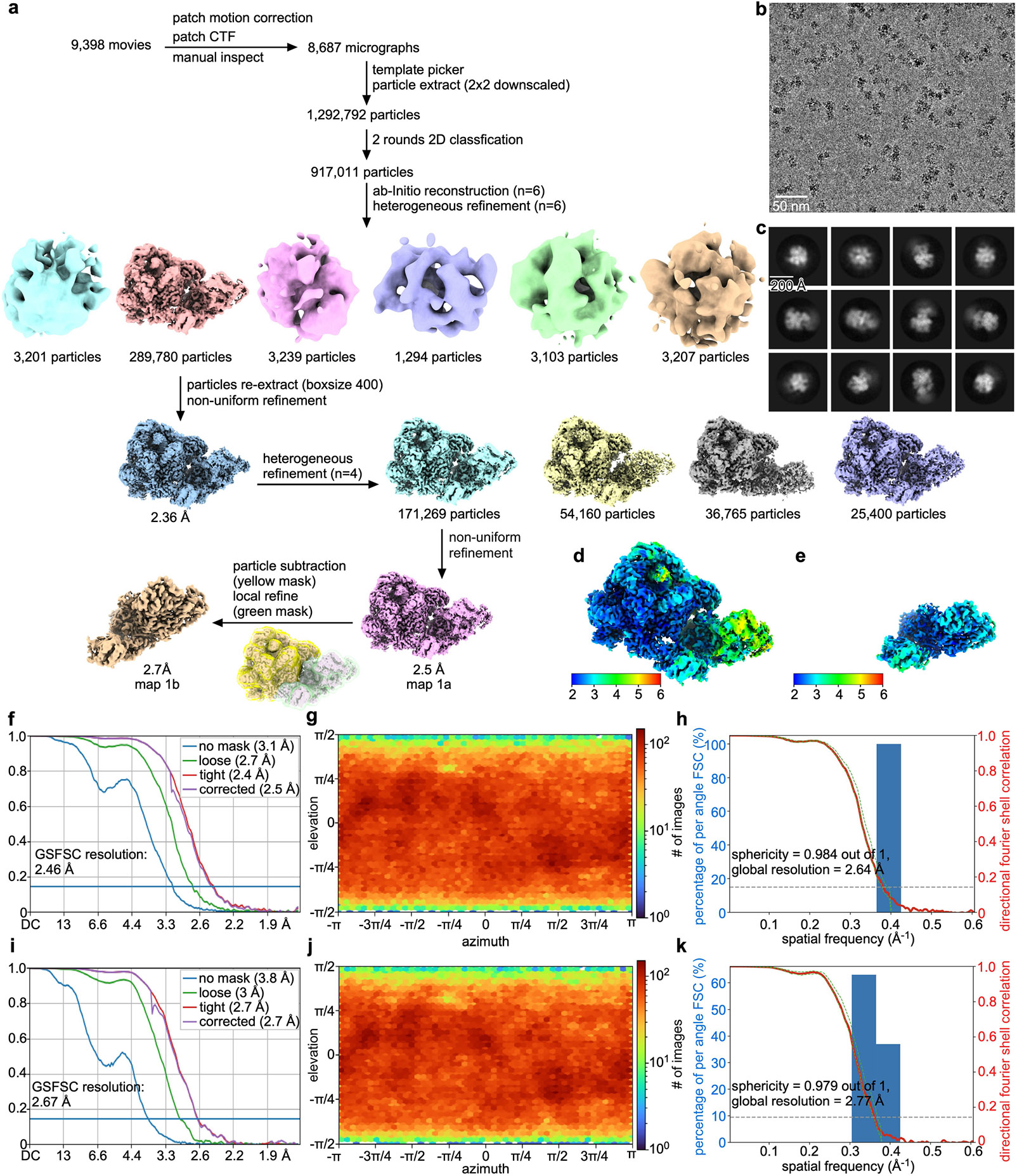
Structure determination: *Tko* FttA(H255A/H591A)–Spt4–Spt5–TEC^44^. **a**, Data processing scheme (Extended Data Table 1). **b**, Representative electron microphotograph. **c**, 2D class averages. **d**,**e**, EM density map coloured by local resolution for global map (map 1a) and locally refined map for FttA, RNAP stalk, and Spt5 KOW (map 1b). **f**–**h**, Fourier-shell correlation (FSC) plot, orientation distribution plot, and 3D FSC plot for map 1a. **i**–**k**, Fourier-shell correlation (FSC) plot, orientation distribution plot, and 3D FSC plot for map 1b.

**Extended Data Fig. 3 ∣:**
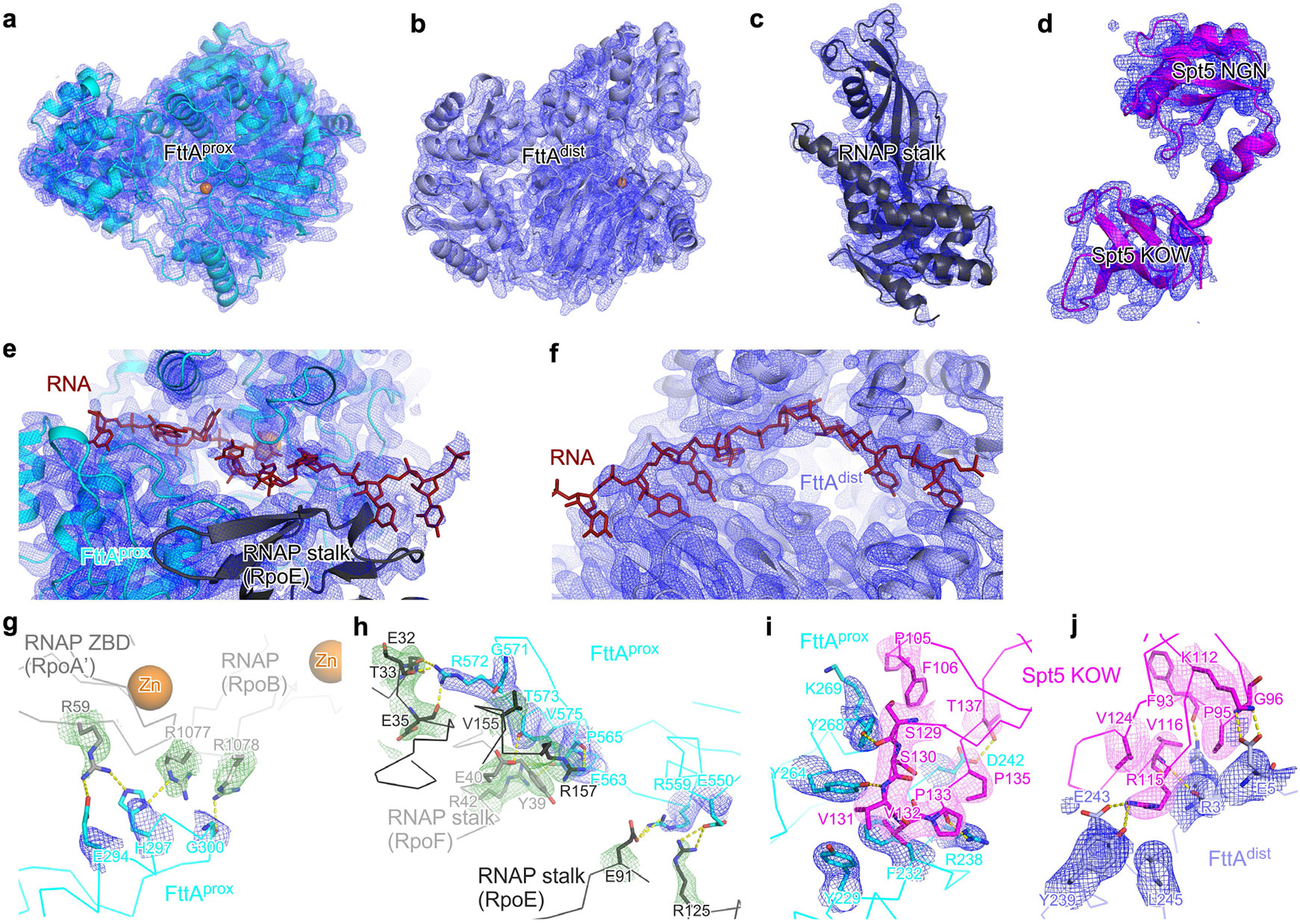
Structure: *Tko* FttA(H255A/H591A)–Spt4–Spt5–TEC^44^. **a**–**j**, Representative EM density (isocontours) and fits (Cα traces for backbones; sticks for sidechains) for FttA^prox^, FttA^dist^, RNAP stalk (RpoE and RpoF), Spt5, RNA interacting with FttA^prox^, RNA interacting with FttA^dist^, interface between FttA^prox^ and RNAP RpoA’ and RpoB, interface between FttA^prox^ and RNAP stalk (RpoE and RpoF), interface between FttA^prox^ and Spt5, and interface between FttA^dist^ and Spt5. Colours as in Fig. 2.

**Extended Data Fig. 4 ∣:**
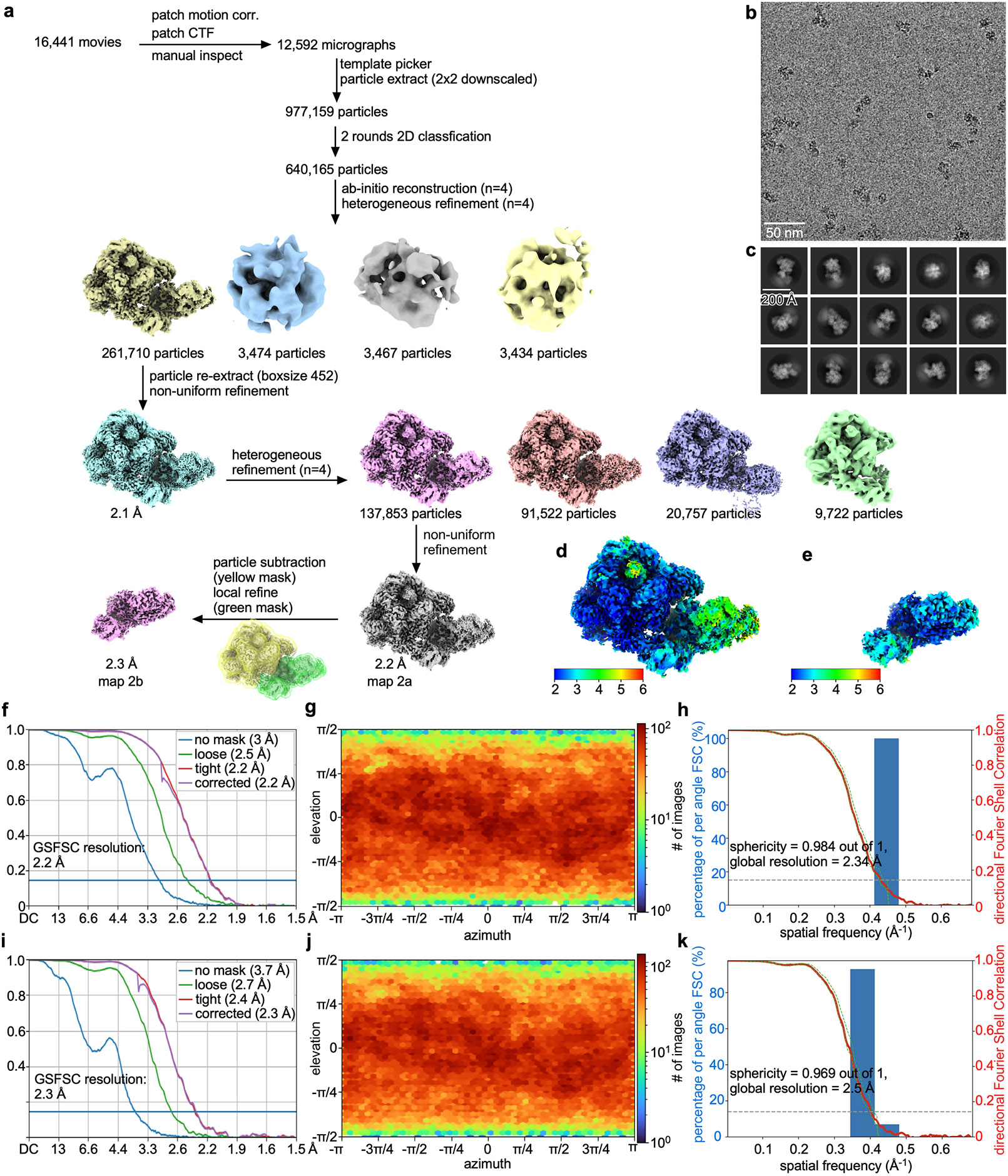
Structure determination: *Tko* FttA(H255A/H591A)–Spt4–Spt5–TEC^52^. **a**, Data processing scheme (Extended Data Table 1). **b**, Representative electron microphotograph. **c**, 2D class averages. **d**,**e**, EM density map coloured by local resolution for global map (map 2a) and locally refined map for FttA, RNAP stalk, and Spt5 KOW (map 2b). **f**–**h**, Fourier-shell correlation (FSC) plot, orientation distribution plot, and 3D FSC plot for map 2a. **i**–**k**, Fourier-shell correlation (FSC) plot, orientation distribution plot, and 3D FSC plot for map 2b.

**Extended Data Fig. 5 ∣:**
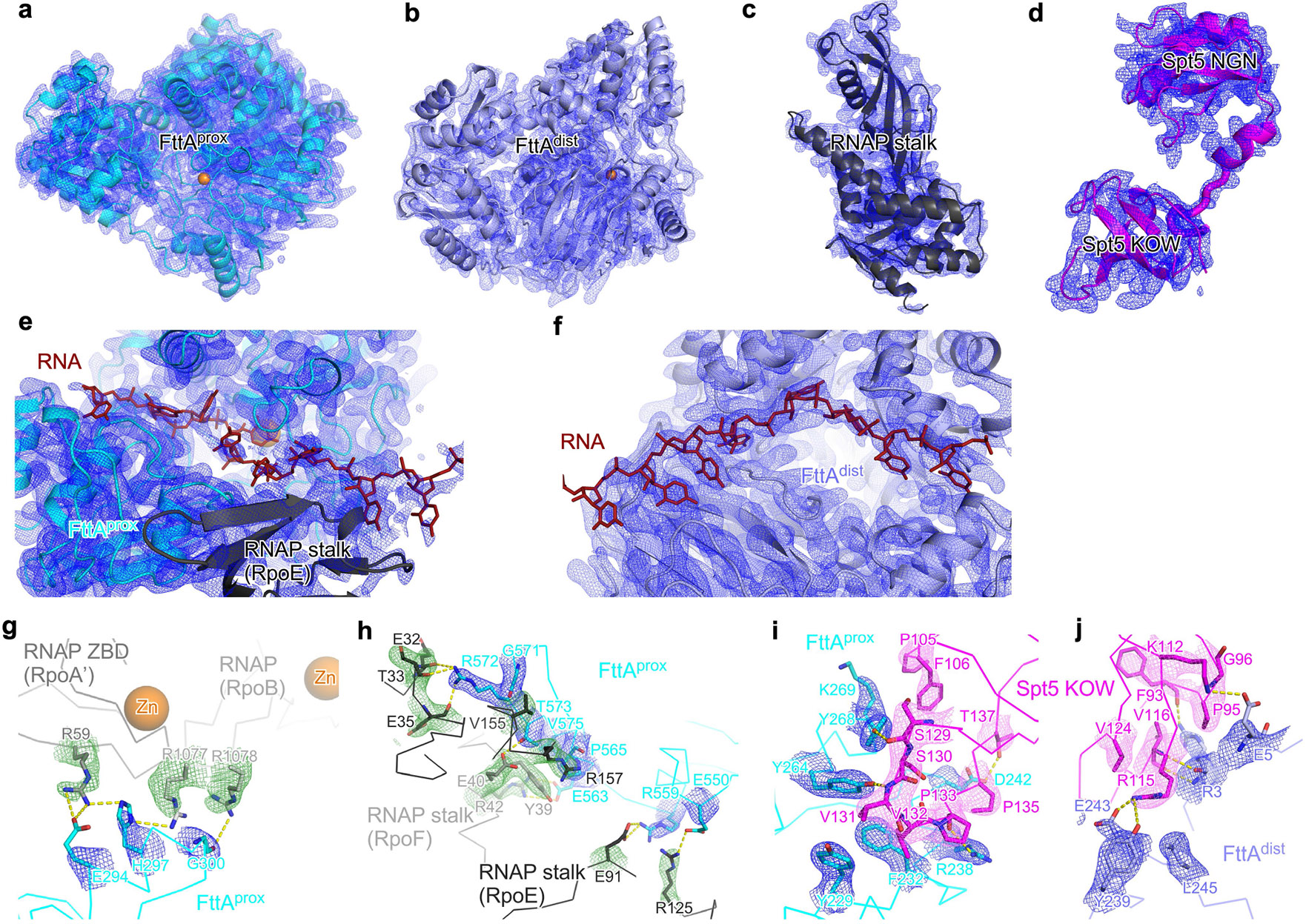
Structure: *Tko* FttA(H255A/H591A)–Spt4–Spt5–TEC^52^. **a**–**j**, Representative EM density (isocontours) and fits (Cα traces for backbones; sticks for sidechains) for FttA^prox^, FttA^dist^, RNAP stalk (RpoE and RpoF), Spt5, RNA interacting with FttA^prox^, RNA interacting with FttA^dist^, interface between FttA^prox^ and RNAP RpoA’ and RpoB, interface between FttA^prox^ and RNAP stalk (RpoE and RpoF), interface between FttA^prox^ and Spt5, and interface between FttA^dist^ and Spt5. Colours as in Fig. 2.

**Extended Data Fig. 6 ∣:**
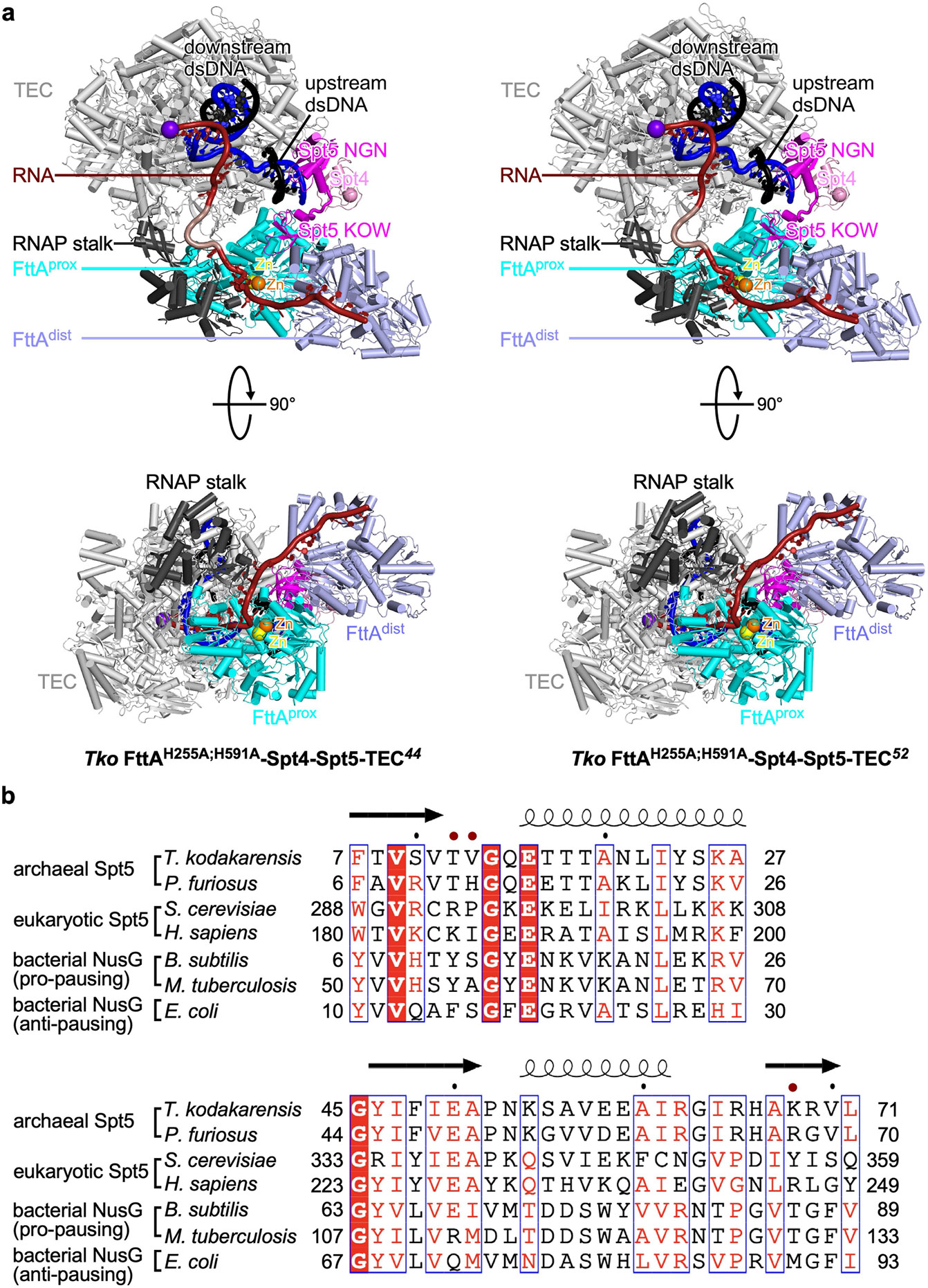
Structure: *Tko* FttA(H255A/H591A)–Spt4–Spt5–TEC^44^ and *Tko* FttA(H255A/H591A)–Spt4–Spt5–TEC^52^. **a**, Comparison of structures *Tko* FttA(H255A/H591A)–Spt4–Spt5–TEC^44^ (left) and *Tko* FttA(H255A/H591A)–Spt4–Spt5–TEC^52^ (right). Colours as in Fig. 2. **b**, Sequence alignment of regions of archaeal Spt5 that contact nontemplate-strand DNA in *Tko* FttA(H255A/H591A)–Spt4–Spt5–TEC^44^ and *Tko* FttA(H255A/H591A)–Spt4–Spt5–TEC^52^ to corresponding regions of yeast and human Spt5, *Bacillus subtilis* and *Mycobacterium tuberculosis* NusG (which have “pro-pausing” activity and which make corresponding protein-DNA interactions^22,23^), and *E. coli* NusG (which has “anti-pausing” activity and which does not make corresponding protein-DNA interactions^21^). Arrows, β-strands; helices, α-helices; red dots, residues that contact nontemplate-strand DNA in *Tko* FttA(H255A/H591A)–Spt4–Spt5–TEC^44^ and *Tko* FttA(H255A/H591A)–Spt4–Spt5–TEC^52^. Boxes denote conserved sequence positions. Colours denote levels of sequence conservation (red fill with white letters, high conservation; red letters, moderate conservation).

**Fig. 2 ∣:**
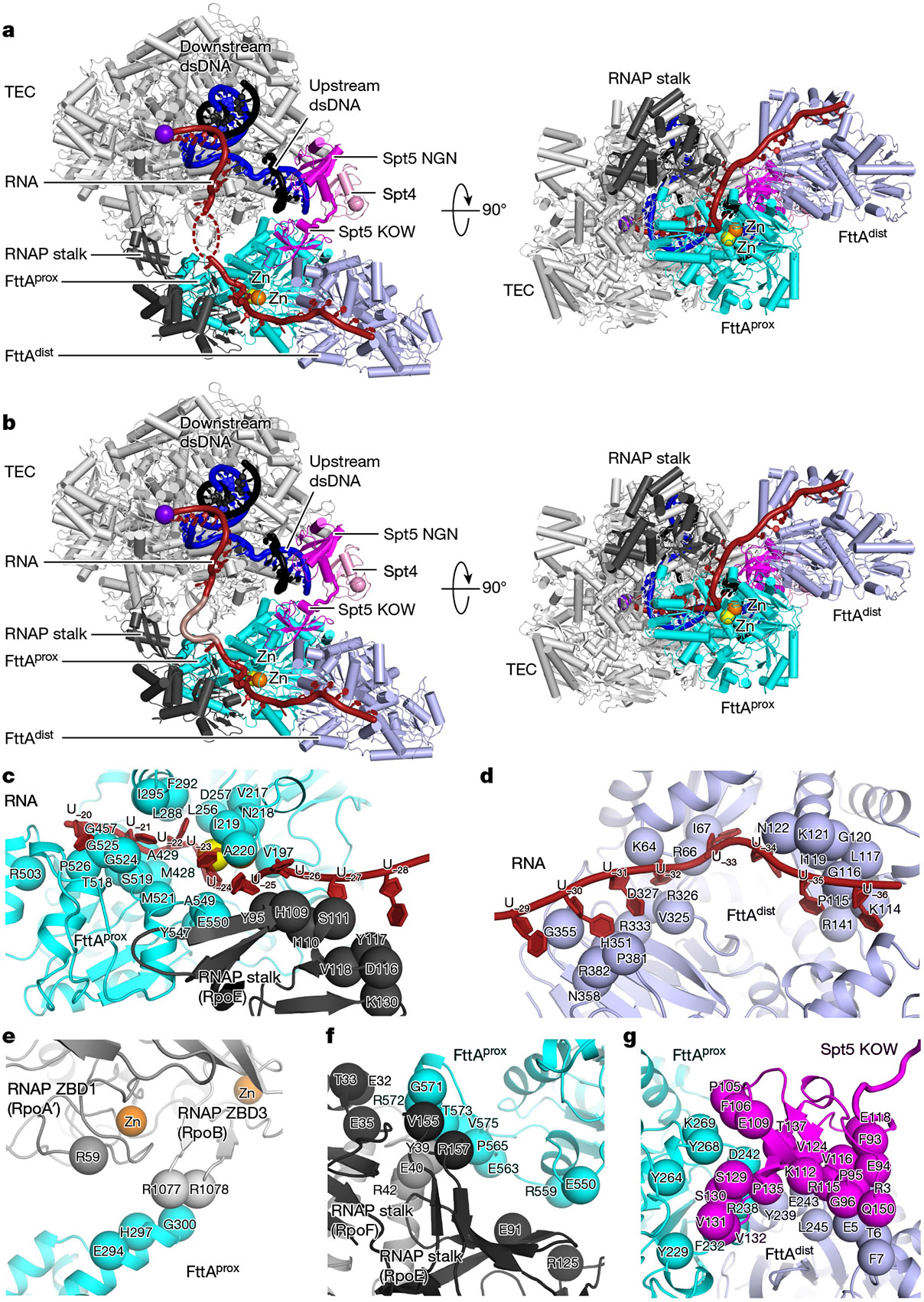
Structure of the FttA pre-termination complex (*Tko* FttA(H255A/H591A)–Spt4–Spt5–TEC^52^). **a**, Cryo-EM structure. Left, view with the TEC RNA-exit channel aligned with the *y* axis, showing passage of RNA through the TEC RNA-exit channel and across FttA^prox^ and FttA^dist^. Right, orthogonal view showing the inferred RNA path across FttA^prox^ and FttA^dist^. FttA^prox^, cyan, with the nucleolytic active-centre Zn^2+^ ion (I) as orange sphere and position of nucleolytic active-centre catalytic Zn^2+^ ion (II) (absent due to H255A/H591A substitution in the FttA derivative used for structure determination; position based on superimposition of PDB 3AF5^5^) as yellow sphere; FttA^dist^, lavender; Spt4, pink; Spt5, magenta; RNAP TEC, grey; RNAP stalk, dark grey; nontemplate-strand DNA, black; template-strand DNA, blue; RNA, brick red. dsDNA, double-stranded DNA. **b**, Cryo-EM structure with inferred full path of RNA through TEC RNA-exit channel, FttA^prox^, and FttA^dist^. RNA segment in central and distal parts of TEC RNA-exit channel, modelled by superimposition and interpolation, is shown in pink. **c**, Protein–RNA interactions of FttA^prox^ and RNAP stalk (RpoE). **d**, Protein–RNA interactions of FttA^dist^. **e**, Protein–protein interactions of FttA^prox^ with RNAP zinc-binding domain 1 (RpoA′ ZBD1) and RNAP zinc-binding domain 3 (RpoB ZBD3). **f**, Protein–protein interactions of FttA^prox^ with RNAP stalk (RpoE and RpoF). **g**, Protein–protein interactions of FttA^prox^ and FttA^dist^ with Spt5 KOW.

We assembled a FttA pre-termination complexes from *Tko* FttA(H255A/H591A), *Tko* Spt5, *Tko* Spt4^17-19^ (the binding partner of Spt5), *Tko* RNAP, and synthetic nucleic acid scaffolds that contained determinants for formation of a TEC and that contained either a 44-nt or a 52-nt RNA oligomer having a U-rich 5′ extension able to serve as an FttA binding site (scaffolds TEC^44^ and TEC^52^, respectively; sequences presented in Extended Data Fig. 1a,b; functional assay for scaffold TEC^44^ shown in Fig. 1c). We then determined 2.5 Å and 2.2 Å resolution structures of the complexes by use of single-particle-reconstruction cryo-EM (FttA(H255A/H591A)–Spt4–Spt5–TEC^44^ and FttA(H255A/H591A)–Spt4–Spt5–TEC^52^; Fig. 2a, Extended Data Figs. 2-6 and Extended Data Table 1).

The structures obtained using the two synthetic nucleic acid scaffolds are superimposable (Extended Data Fig. 6). In each case, the structure shows that a *Tko* FttA dimer (comprising protomer FttA^prox^ located proximal to the TEC and protomer FttA^dist^ located distal to the TEC) interacts with the *Tko* TEC at the mouth of the TEC RNA-exit channel, enabling interactions of the FttA dimer with nascent RNA emerging from the RNA-exit channel (Fig. 2a). The structure further shows that *Tko* Spt5 bridges the TEC and FttA, with the Spt5 NGN domain interacting with its binding partner Spt4 and the TEC, and with the Spt5 KOW domain interacting with FttA^prox^ and FttA^dist^ (Fig. 2a). *Tko* Spt5 NGN interacts with the largest and second-largest subunits of RNAP in the same manner as for NusG NGN in structures of bacterial TECs^21-23^ and for Spt5 NGN in structures of eukaryotic TECs^24^. *Tko* Spt5 NGN additionally makes interactions—through non-conserved regions of NGN involving Spt5 residues 12, 13, and 68—with the backbone of nontemplate-strand DNA immediately upstream of the transcription bubble, reminiscent of interactions made by NusG NGN in structures of bacterial TECs from bacterial species in which NusG has ‘pro-pausing’ activity^22,23^ (Extended Data Fig. 6b). The structures show the TEC in a post-translocated state. The structures resolve 12 nt of nascent RNA in the TEC RNA–DNA hybrid and proximal part of the TEC RNA-exit channel and resolve 17 nt of nascent RNA interacting with FttA^prox^ and FttA^dist^, but the structures do not resolve RNA nucleotides in the central and distal parts of the TEC RNA-exit channel (Fig. 2a and Extended Data Figs. 3e,f and 5e,f). On the basis of the position of the FttA-dependent endonucleolytic site mapped in Fig. 1c, and on the position of the FttA^prox^ endonucleolytic and exonucleolytic active centre in the structures (Fig. 2a and Extended Data Figs. 3e and 5e), we infer that 7 nt of nascent RNA are present in the central and distal parts of the TEC RNA-exit channel but are disordered—existing as an unresolved ensemble of different compacted conformational states—as has been observed previously in structures of TECs having more than 7 nt of RNA in the RNA-exit channel^25,26^.

Although 7 nt of nascent RNA in the central and distal parts of the TEC RNA-exit channel are not resolved in the structures, we are able to model 6 of those 7 nt of nascent RNA by superimposing a published structure of a TEC containing 6 nt of RNA in the central and distal parts of the RNA-exit channel (Protein Data Bank (PDB) 6VYR)^25^. Connection of the superimposed RNA segment to the observed RNA segments, interpolating 1 nt between the superimposed RNA segment and the observed RNA segment interacting with FttA^prox^ and FttA^dist^, provides a model of the full path of the nascent RNA (Fig. 2b).

**Extended Data Fig. 7 ∣:**
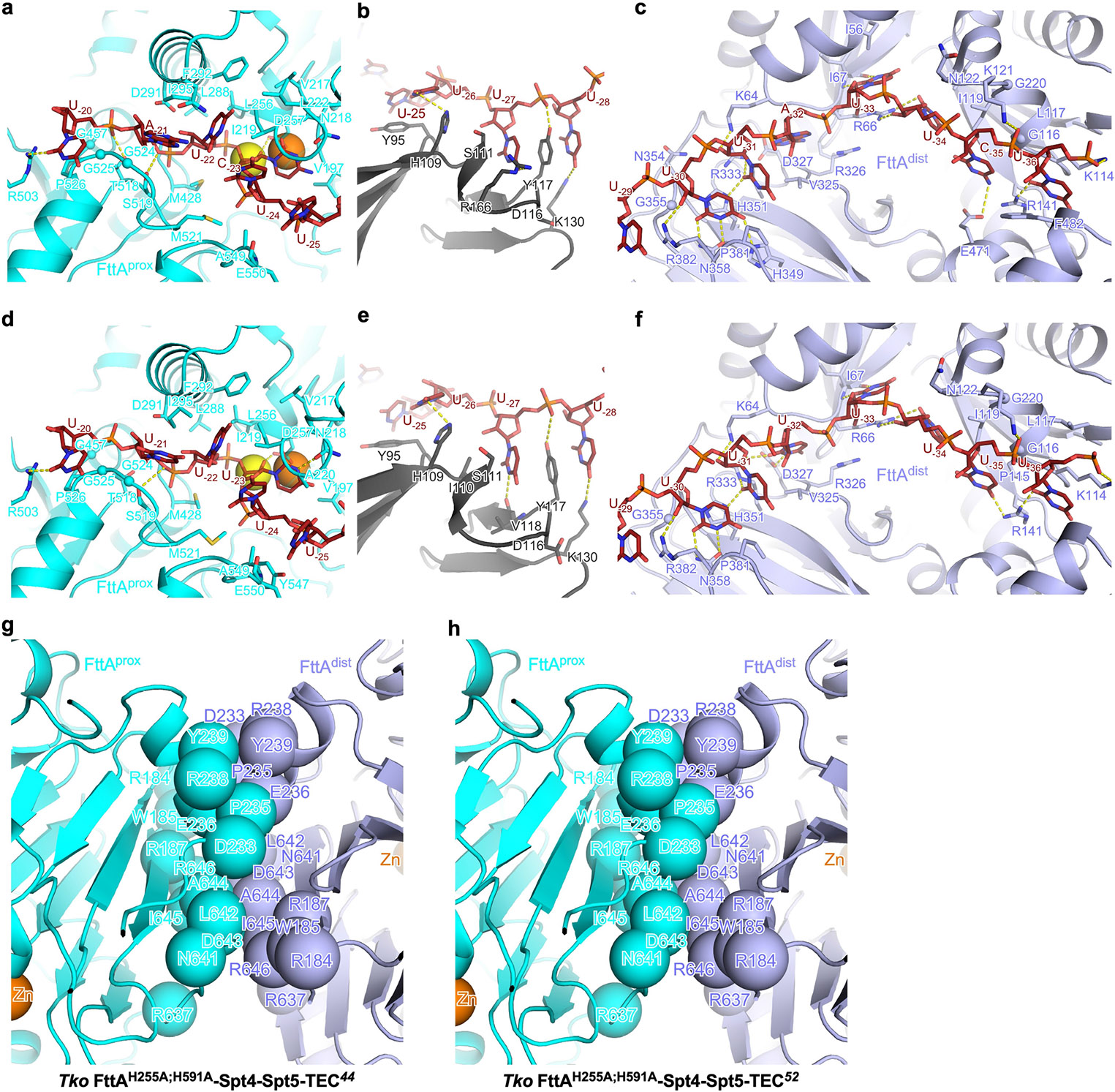
Protein-RNA interactions by FttA and dimerization interface of FttA in *Tko* FttA(H255A/H591A)–Spt4–Spt5–TEC^44^ and *Tko* FttA(H255A/H591A)–Spt4–Spt5–TEC^52^. **a**–**f**, Details of protein-RNA interactions by FttA^prox^, RNAP stalk, and FttA^dist^ in *Tko* FttA(H255A/H591A)–Spt4–Spt5–TEC^44^ (panels **a**–**c**) and *Tko* FttA(H255A/H591A)–Spt4–Spt5–TEC^52^ (panels **d**–**f**). Colours as in Fig. 2. **g**,**h**, Interface between FttA^prox^ and FttA^dist^ in *Tko* FttA(H255A/H591A)–Spt4–Spt5–TEC^44^ (panel **g**) and *Tko* FttA(H255A/H591A)–Spt4–Spt5–TEC^52^ (panel **h**). Colours as in Fig. 2.

In the resulting model of the full path of the nascent RNA, the RNA proceeds directly from the TEC RNA-exit channel, through the FttA^prox^ active-centre groove, to the FttA^prox^ nucleolytic active centre (Fig. 2b,c and Extended Data Fig. 7a,d). RNA then proceeds from the FttA^prox^ nucleolytic active centre, through a continuation of the FttA^prox^ active-centre groove, and along a surface of the RNAP stalk, to the FttA^dist^ KH1 and KH2 domains (Fig. 2b-d and Extended Data Fig. 7b,c,e,f). Six nucleotides of RNA, positioned 20–25 nt from the RNA 3′ end in a TEC in the post-translocated state, interact with FttA^prox^ (RNA nucleotides −20 to −25, assigning the RNA 3′ nucleotide as −1; Fig. 2b,c and Extended Data Fig. 7a,d); 4 nt of RNA, positioned 25–28 nt from the RNA 3′ end in a TEC in the post-translocated state, interact with the RNAP stalk (RNA nucleotides −25 to −28; Fig. 2b,c and Extended Data Fig. 7b,e); and 8 nt of RNA, positioned 29–36 nt from the RNA 3′ end in a TEC in the post-translocated state, interact with FttA^dlst^ (RNA nucleotides −29 to −36; Fig. 2b,d and Extended Data Fig. 7c,f). RNA nucleotides −22 and −23 engage the FttA^prox^ nucleolytic active centre, with the 5′-phosphate of RNA nucleotide −22 and the 3′-hydroxyl of RNA nucleotide −23 positioned close to the catalytic Zn^2+^ ions, poised for endonucleolytic cleavage 22 nt from the RNA 3′ end (Fig. 2b,c and Extended Data Fig. 7a,d).

The protein–RNA interactions made by FttA in the structures of the FttA-dependent pre-termination complex are similar to those predicted based on a crystal structure of MβL and β-CASP domains bound to RNA (PDB 3IE1) and a crystal structure of the KH1 domain of FttA bound to RNA^5-7^ (PDB 3AF6). The protein–RNA interactions between FttA^prox^ and RNA nucleotide −23—one of the two RNA nucleotides that engage the FttA^prox^ nucleolytic active centre—involve the RNA base and include steric constraints and a hydrogen bond with the RNA base that appear likely to confer selectivity for U (Extended Data Fig. 7a,d). The protein–RNA interactions between the FttA^dist^ KH1 and KH2 domains and RNA nucleotides −30 to −32 and −34 to −36 all involve RNA bases, all include hydrogen bonds with RNA bases, and all appear likely to confer selectivity for U (Extended Data Fig. 7c,f). The observation that FttA^dist^ KH1 and KH2 domains interact with six RNA nucleotides in a manner likely to confer selectivity for U is consistent with published biochemical results indicating that the FttA KH1 and KH2 domains are an important determinant of selectivity for U^3^.

The observed protein–RNA interactions indicate that an FttA-dependent termination site—an ‘FttA-dependent terminator’—comprises RNA nucleotides −1 to −36 relative to the 3′ end of nascent RNA in a post-translocated TEC, optimally contains U at position −23, and optimally contains U-rich RNA at positions −30 to −36. The observed protein–RNA interactions further indicate that the cleaved RNA products generated during FttA-dependent termination optimally contain U at position −1 relative to the cleavage-generated 3′ end and optimally contain U-rich RNA at positions −9 through −14 relative to the cleavage-generated 3′ end, consistent with experimentally determined 3′-end sequences of FttA-terminated RNAs^2,3^.

The nascent RNA in the FttA-dependent pre-termination complex interacts with the opposite faces of FttA^prox^ and FttA^dist^, interacting with the face of FttA^prox^ that contains the active-centre groove and the nucleolytic catalytic centre, and interacting with the face of FttA^dist^ that does not contain the active-centre groove and the nucleolytic catalytic centre, and, instead, contains the KH1 and KH2 domains (Fig. 2c,d). We infer that FttA^prox^ and FttA^dist^ have distinct functional roles. FttA^prox^ has the key role, carrying out the endonucleolytic and exonucleolytic cleavage that drive termination, and FttA^dist^ has a supporting role, providing an extension of the protein–RNA interaction surface that enables additional interactions with the nascent RNA, enhanced sequence-selectivity for U-rich regions in nascent RNA, and enhanced stability of the pre-termination complex. The inferred involvement of both protomers of the FttA dimer in FttA–RNA interactions in the FttA pre-termination complex is consistent with, and accounts for, the observation that dimerization of FttA enhances the RNA-cleavage activity of FttA^3,7^.

The protein–protein interface that connects TEC and Spt5 to FttA is large (1,895 Å^2^ buried surface area; Fig. 2a,b,e-g), suggesting that the translational and rotational orientation of TEC and Spt5 relative to FttA is fixed or nearly fixed, and likely remains fixed or nearly fixed, during nucleolytic cleavage and termination by FttA^prox^. The protein–protein interface has three parts: (1) a set of interactions between the largest and second-largest subunits of RNAP (RpoA′ and RpoB) and FttA^prox^, comprising interactions by RpoA′ zinc-binding domain 1 (ZBD1) and RpoB zinc-binding domain 3 (ZBD3) (Fig. 2e; 252 Å^2^ buried surface area); (2) an interaction between the RNAP stalk (RpoE and RpoF) and FttA^prox^ (Fig. 2f; 619 Å^2^ buried surface area); and (3) interactions between Spt5 KOW and FttA^prox^ and between Spt5 KOW and FttA^dist^ (Fig. 2g; 1,024 Å^2^ buried surface area).

**Extended Data Fig. 8 ∣:**
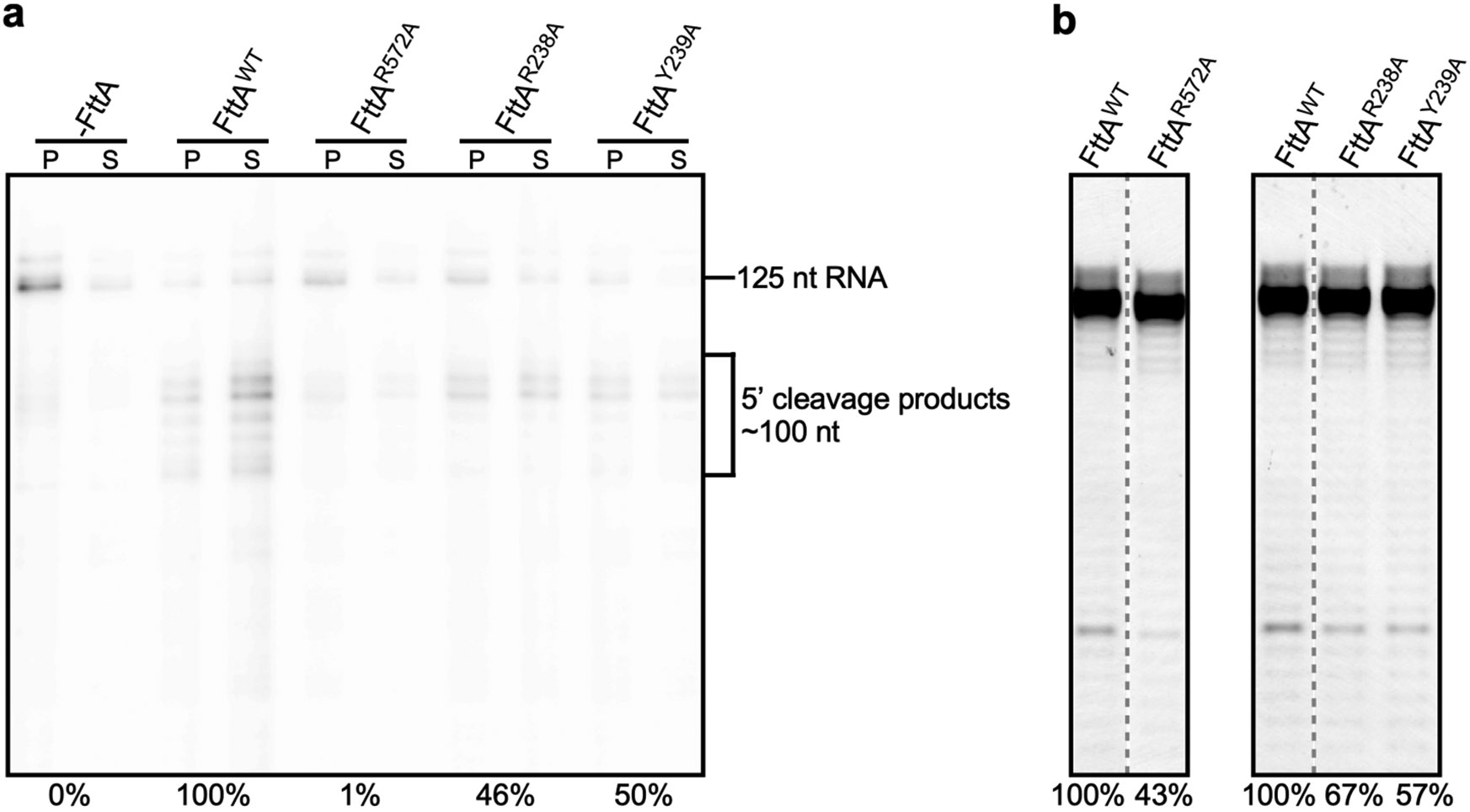
Protein-protein interactions by FttA: effects of alanine substitution of FttA residues that contact RNAP or Spt5 in *Tko* FttA(H255A/H591A)–Spt4–Spt5–TEC^44^ and *Tko* FttA(H255A/H591A)–Spt4–Spt5–TEC52^52^. **a**, Effects of alanine substitution on FttA-dependent transcription termination and RNA cleavage, assessed in release assays with bead-immobilized promoter-generated TECs containing ^32^P-5’-end-labelled RNA, detecting retained or released RNA by storage-phosphor scanning (methods as in ref. 1 and Fig. 1b). P, pellet fraction; S, supernatant fraction. Top, gel image. Bottom, normalized efficiencies of FttA-dependent RNA cleavage [((RNA-cleavage efficiency)/(RNA-cleavage efficiency with wild-type FttA))100%]. Assays were performed twice, with consistent results obtained. For gel source data, see Supplementary Fig. 1. **b**, Effects of alanine substitution on FttA-dependent RNA cleavage, assessed in assays with TECs assembled on synthetic nucleic-acid scaffolds containing 44 nt fluorescein-5’-end-labelled nascent RNA, detecting intact and cleaved RNA by x/y fluorescence scanning (methods as in Fig. 1c). Top, gel image. Bottom, normalized efficiencies of FttA-dependent RNA cleavage [((RNA-cleavage efficiency)/(RNA-cleavage efficiency with wild-type FttA))100%]. Assays were performed twice, with consistent results obtained. For gel source data, see Supplementary Fig. 1.

The involvement of the RNAP stalk and Spt5 in protein–protein interactions with FttA in our structures is consistent with, and accounts for, the observation that the RNAP stalk and Spt5 are essential for FttA-dependent termination^1^. Alanine substitutions of FttA residue R572 in the FttA^prox^–RNAP interface (Fig. 2f and Extended Data Figs. 3h and 5h), FttA residue R238 in the FttA^prox^–Spt5 interface (Fig. 2g and Extended Data Figs. 3i and 5i), and FttA residue Y239 in the FttA^dist^–Spt5 interface (Fig. 2g and Extended Data Figs. 3j and 5j) result in defects in FttA-dependent RNA cleavage, further confirming the functional importance of the interactions in our structures (Extended Data Fig. 8).

The dimerization interface between FttA^prox^ and FttA^dist^ also is large (Extended Data Fig. 7g,h; 1,002 Å^2^ buried surface area). The dimerization interface observed in our structure of the FttA pre-termination complex is identical to the dimerization interface observed in crystal structures of FttA in the absence of other factors^6,7^.

## Mechanism of FttA-dependent termination

The orientation of FttA^prox^ relative to the TEC in the structure (Fig. 2b), and the position and orientation of the FttA^prox^ nucleolytic catalytic site relative to the TEC in the structure (Fig. 2b), support the proposal^1-3^ that FttA mediates termination by performing endonucleolytic cleavage of the RNA followed by processive 5′→3′ exonucleolytic cleavage of the RNA, resulting in 5′→3′ translocation of FttA on the RNA toward the TEC, application of mechanical force to the TEC, disruption of the TEC, and termination. The key premise of this proposed is that each step of processive 5′→3′ exonucleolytic activity by FttA necessarily results in 5′→3′ translocation of FttA relative to RNA, and, in the context of the extensive and probably fixed or nearly fixed protein–protein interface between FttA and the TEC, necessarily applies mechanical force through the RNA to the TEC.

## Mechanisms of factor-dependent termination

Our structure of the archaeal FttA pre-termination complex defines the relationship of archaeal factor-dependent termination to bacterial and eukaryotic factor-dependent termination.

**Fig. 3 ∣:**
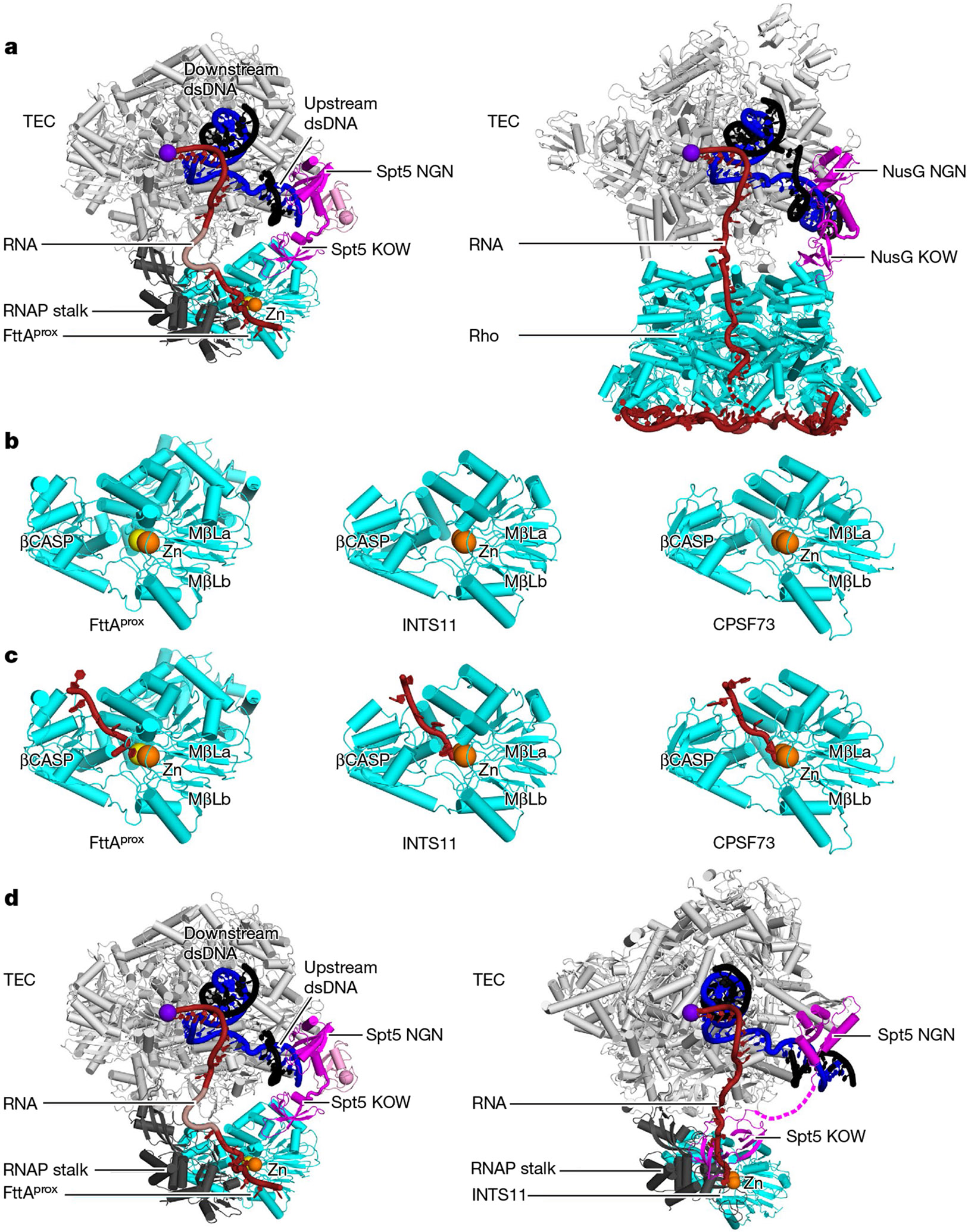
Structural analogy of factor-dependent pre-termination complexes in bacteria and archaea, and structural homology of factor-dependent pre-termination complexes in archaea and eukaryotes. **a**, Comparison of archaeal FttA-dependent pre-termination complex (left; Fig. 2) and bacterial Rho-dependent pre-termination complex (right; PDB 8E6W and PDB 8E6X)^20^. In the left panel, FttA^prox^ KH1 and KH2 domains and FttA^dist^ are omitted for clarity. Rho, cyan; NusG, magenta. Other colours as in Fig. 2b. **b**, Comparison of MβL and β-CASP domains of archaeal FttA^prox^ (Fig. 2), eukaryotic INTS11 (PDB 7YCX)^9^, and eukaryotic CPSF73 (PDB 6V4X)^27^. MβL and β-CASP, cyan; nucleolytic active-centre Zn^2+^ ions, orange and yellow spheres. **c**, Comparison of RNA-bound MβL and β-CASP domains of FttA^prox^ (Fig. 2), INTS11 (PDB 7YCX)^9^, and CPSF73 (PDB 6V4X)^27^. RNA segment 3′ to nucleolytic active centre, red. Other colours as in **b**. **d**, Comparison of archaeal FttA-dependent pre-termination complex (left; Fig. 2) and eukaryotic INT-dependent pre-termination complex (right; PDB 7YCX)^9^. In the left panel, FttA^prox^ KH1 and KH2 domains and FttA^dist^ are omitted for clarity. In the right panel, INT and PPA2 subunits other than INTS11, INTS11 CTD, RPB1 CTD, NELF, Spt4 and Spt5 KOW domains other than the N-terminal domain and KOW4 are omitted for clarity. INTS11, cyan; connector between Spt5 NGN and Spt5 KOW4, dashed magenta line. Other colours as in Fig. 2.

The relationship of archaeal FttA-dependent termination to bacterial Rho-dependent termination is a relationship of analogy in the absence of homology. FttA bears no sequence or structural relationships with Rho (Fig. 3a). However, FttA^prox^ interacts with the mouth of the archaeal TEC RNA-exit channel, enabling direct delivery of RNA to the FttA^prox^ catalytic centre in a manner that is highly similar to the manner in which Rho interacts with the mouth of the bacterial TEC RNA-exit channel, enabling direct delivery of RNA to the Rho catalytic centre (Fig. 3a). In addition, Spt5 bridges FttA^prox^ and the archaeal TEC in a manner that is highly similar to how the bacterial Spt5 homologue NusG bridges Rho and the bacterial TEC (Fig. 3a).

The relationship of archaeal FttA-dependent termination to eukaryotic factor-dependent termination is one of homology. The structures of the MβL and β-CASP domains of FttA^prox^ in the FttA pre-termination complex are superimposable on the structures of the MβL and β-CASP domains of eukaryotic INTS11^8-10^, the RNA-cleavage subunit of the 14-subunit, 1.6 MDa INT complex^8-12^, and of eukaryotic CPSF73^27^, the RNA-cleavage subunit of a 21-subunit, 1.8 MDa complex CPSF complex^13-16^ (Fig. 3b). In addition, the inferred protein–RNA interactions made by the MβL and β-CASP domains of FttA^prox^ in the FttA pre-termination complex are superimposable on those made by eukaryotic INTS11 and eukaryotic CPSF73^8-10,27^ (Fig. 3c). Most important, the translational and rotational orientation of archaeal FttA^prox^ relative to the archaeal TEC and Spt5 KOW are highly similar to those of eukaryotic INTS11 and Spt5 KOW4 in published structures of the 14-subunit, 1.6 MDa human INT complex and Spt5 bound to human Pol II^8-10^ (Fig. 3d).

Integrator and CPSF each contains a pseudodimer of two FttA homologues: a first FttA homologue that possesses endoribonucleolytic and exoribonucleolytic activity (INTS11 and CPSF73 in Integrator and CPSF, respectively), and a second FttA homologue that does not possess endoribonucleolytic and exoribonucleolytic activity (INTS9 and CPSF100 in Integrator and CPSF, respectively)^8-10,12,13^. The presence of pseudodimers of catalytically active and catalytically inactive FttA homologues in Integrator and CPSF is reminiscent of the presence of a dimer of catalytically relevant FttA^prox^ and catalytically irrelevant FttA^dist^ in the FttA-dependent pre-termination complex and raises the possibility that the catalytically active FttA homologue, like FttA^prox^, participates both in RNA binding and RNA cleavage, and the catalytically inactive FttA homologue, like FttA^dist^, participates only in RNA binding. However, the relative orientation of the two FttA homologues in Integrator and CPSF differs from that of FttA^prox^ and FttA^dist^ in the FttA-dependent pre-termination complex. Additional work will be required to assess possible functional roles of the catalytically inactive FttA homologues in Integrator and CPSF.

**Extended Data Fig. 9 ∣:**
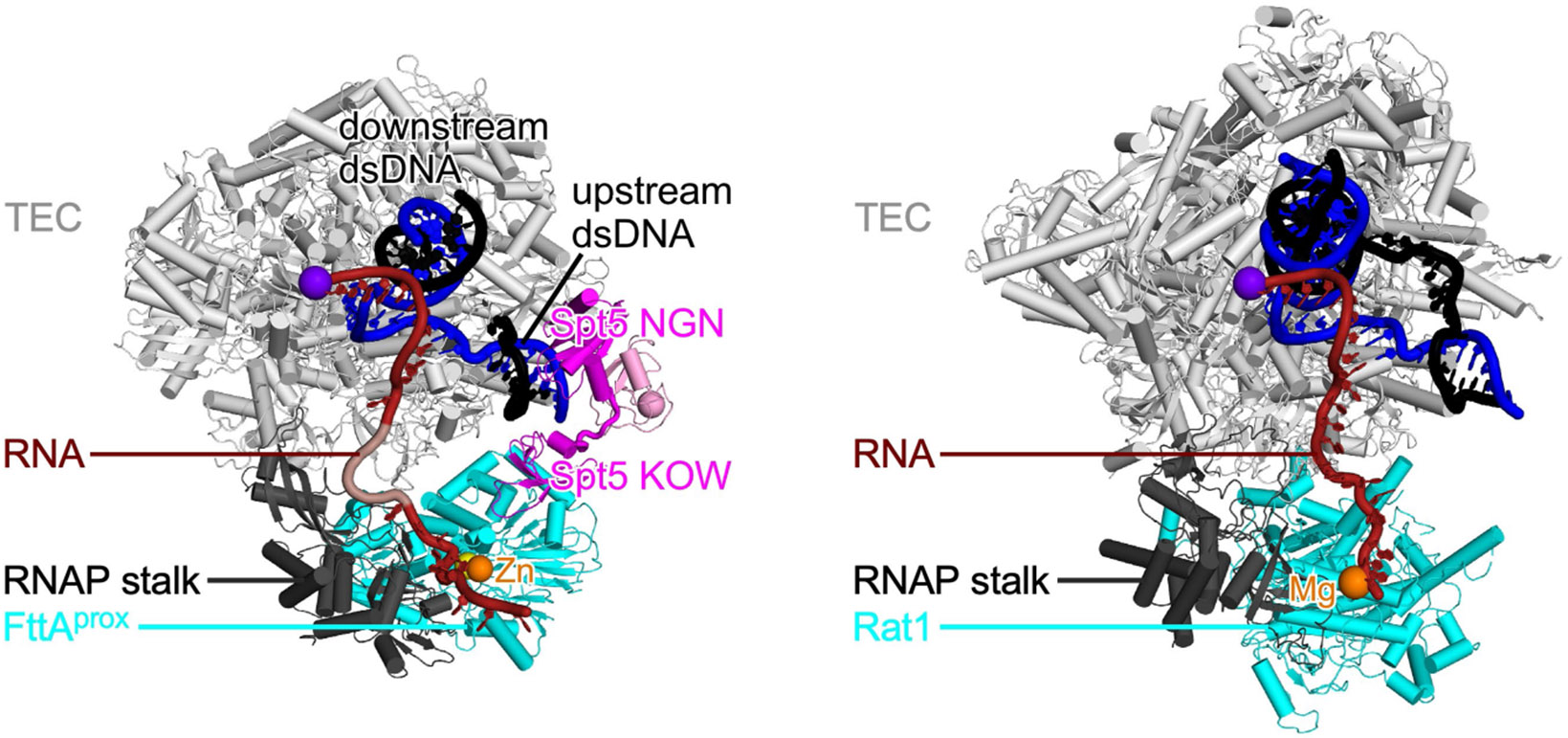
Structural relationship between archaeal FttA-dependent pre-termination complex and eukaryotic XRN2-dependent termination complex. Comparison of archaeal FttA-dependent pre-termination complex (left; Fig. 2) and yeast XRN2 (Rat1)-dependent termination complex (right; PDB 8JCH)^37^. In left panel, FttA^prox^ KH1-KH2 domains and FttA^dist^ are omitted for clarity. In right panel, Rai1 is omitted for clarity. Rat1, cyan. Other colours as in Fig. 2.

**Fig. 4 ∣:**
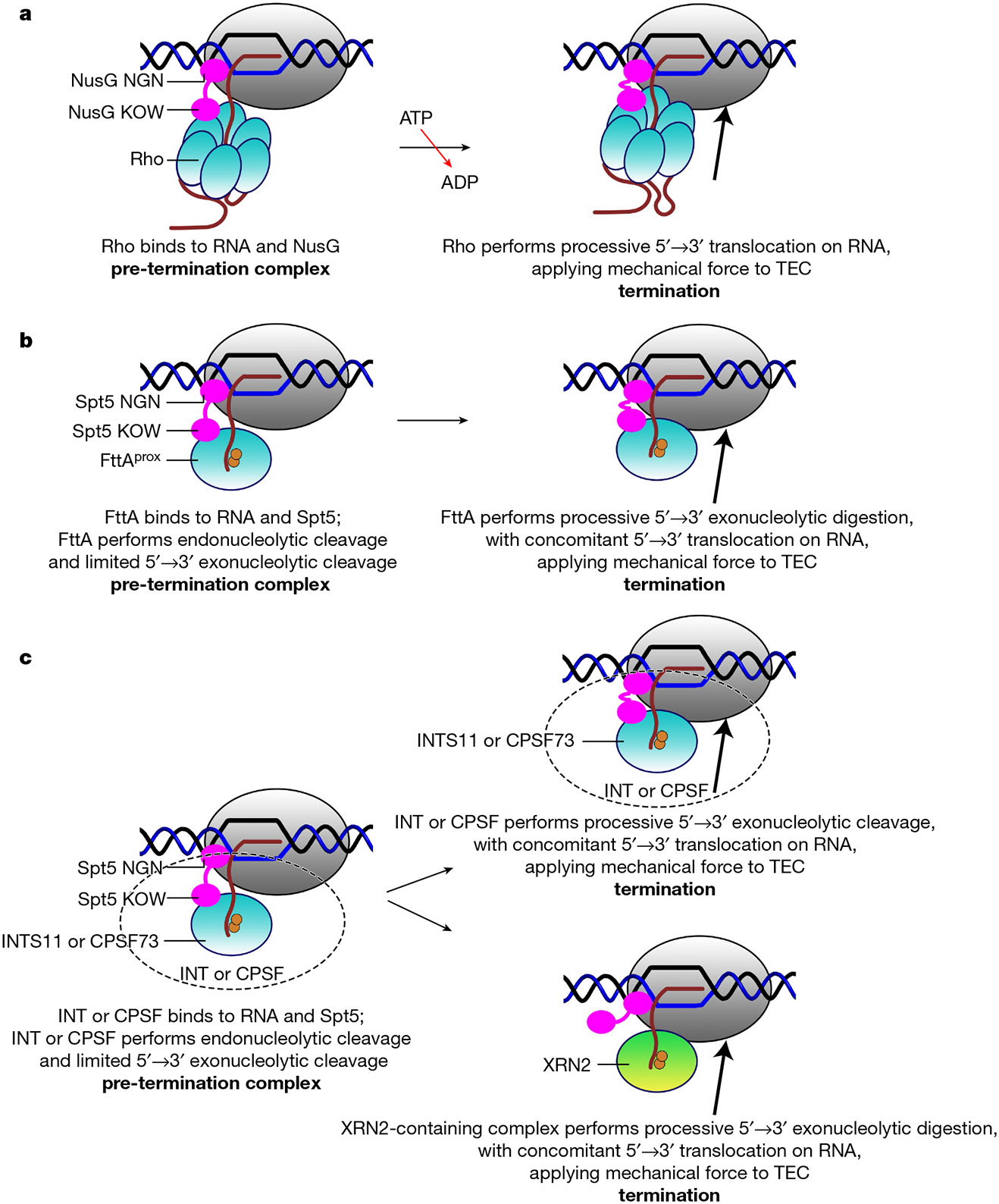
Mechanisms of bacterial, archaeal, and eukaryotic factor-dependent termination. **a**, Bacterial Rho-dependent termination^20^. **b**, Archaeal FttA-dependent termination (Figs. 2 and 3). **c**, Eukaryotic INT-dependent termination (INTS11 as FttA homologue) and CPSF-dependent termination (CPSF73 as FttA homologue)^8-16,30-38^. The top right pathway is thought to predominate for INT-dependent termination^14,35^; the bottom right pathway is thought to predominate for CPSF-dependent termination^13-16,29-36^. Bold text shows the complexes formed by the indicated reactions.

Figure 4 summarizes the mechanisms of factor-dependent termination in the three domains of life: bacteria, archaea, and eukaryotes. In bacterial factor-dependent termination, the RNA translocase Rho binds to RNA emerging from a TEC, stabilized by a bridging interaction by NusG, yielding a pre-termination complex (Fig. 4a, left); Rho then performs ATP-hydrolysis-dependent 5′→3′ translocation on the RNA, thereby applying mechanical force to the TEC and triggering termination^20^ (Fig. 4a, right). In archaeal FttA-dependent transcription termination, the ribonuclease FttA binds to RNA emerging from a TEC, stabilized by a bridging interaction by Spt5, and FttA performs endonucleolytic cleavage and limited 5′→3′ exonucleolytic cleavage of the RNA, yielding a pre-termination complex (Fig. 4b, left); FttA then performs processive 5′→3′ exonucleolytic cleavage of the RNA and concomitant 5′→3′ translocation on the RNA, thereby applying mechanical force on TEC and triggering termination (Fig. 4b, right). In eukaryotic INT- or CPSF-dependent transcription termination, an INTS11- or CPSF73-containing multisubunit, megadalton complex binds to RNA emerging from a TEC, stabilized by a bridging interaction by Spt5, and INTS11 or CPSF73 performs endonucleolytic cleavage and limited 5′→3′ exonucleolytic cleavage of the RNA, forming a pre-termination complex (Fig. 4c, left); then either the INTS11- or CPSF73-containing complex, or an XRN2-containing complex (Rat1-containing complex in yeast), performs processive 5′→3′ exonucleolytic cleavage of the RNA, and concomitant 5′→3′ translocation on the RNA, thereby applying mechanical force to the TEC and triggering termination^8-16,28-38^ (Fig. 4c, right and Extended Data Fig. 9). Available evidence suggests that the mechanism shown in Fig. 4c, top right predominates for INT-mediated termination^14,35^, and the mechanism shown in Fig. 4c, bottom right predominates for CPSF-mediated termination^13-16,29-36^.

The mechanisms proposed in Fig. 4 suggest a fundamental mechanistic unity of factor-dependent termination across the three domains of life: bacteria, archaea, and eukaryotes. In each case, factor-dependent termination is proposed to involve 5′→3′ translocation on RNA by a termination factor that interacts with RNA emerging from the TEC RNA-exit channel, 5′→3′ translocation on RNA by the termination factor is proposed to apply mechanical force to the TEC, and the application of mechanical force to the TEC is proposed to trigger termination. Consistent with these proposals, single-molecule force measurements show that the application of mechanical force through RNA to a TEC can trigger termination^39^. The specific reactions through which the application of mechanical force through RNA to a TEC triggers termination, inhibiting RNA extension and predisposing the TEC to dissociation, remain to be elucidated. It is likely that these reactions include one or more of: (1) forward translocation of the TEC without nucleotide addition (hypertranslocation model)^39-43^; (2) extraction of RNA from the TEC (RNA extraction model)^39,42,43^; and (3) reorganization of TEC structure (allosteric model)^42-45^.

While this work was under review for publication, Zeng et al.^37^ and Yanagisawa et al.^38^ reported cryo-EM structures of termination complexes containing the exoribonuclease XRN2 (yeast Rat1; Fig. 4c, right bottom). The exoribonuclease XRN2 has no sequence or structural similarity to FttA, INTS11, or CPSF73; XRN2 lacks the RNA-sequence-recognition and endoribonuclease activities possessed by FttA, INTS11, and CPSF73; and XRN2 does not perform the termination-site recognition and RNA 3′-end formation reactions performed by FttA, INTS11, and CPSF73 (Fig. 4b,c, left), instead participating only in the TEC-disruption reaction after ‘hand off’ from INT or CSF^29-36^ (Fig. 4c, bottom right). Nevertheless, the structures of Zeng et al.^37^ and Yanagisawa et al.^38^ show that XRN2 interacts with the TEC at the mouth of the TEC RNA-exit channel in XRN2-dependent termination complexes in a manner that is highly analogous to FttA in an FttA-dependent pre-termination complex (Extended Data Fig. 9), and suggest an RNA-translocation-dependent, mechanical-force-dependent mechanism for XRN2-dependent termination that is consistent with our proposed RNA-translocation-dependent, mechanical-force-dependent, unified mechanism of factor-dependent termination in bacteria, archaea, and eukaryotes (Fig. 4).

## Discussion

In bacteria and archaea, there is no nuclear membrane, and transcription and translation occur in the same cellular compartment and can be physically coupled^25,26,46-49^. In bacteria, Rho not only mediates factor-dependent transcription termination, but also mediates quality control of transcription–translation coupling, detecting lapses in transcription–translation coupling, such as losses of transcription–translation coupling during translational arrest, and resolving lapses in transcription–translation coupling through transcription termination^20,46-49^. The structures of the *E. coli* Rho-dependent pre-termination complex^20^ and the *E. coli* NusG-coupled transcription–translation complex^25,46^ indicate that the mechanism of Rho-dependent quality control of transcription–translation coupling relies on a simple steric-exclusion mechanism: namely, the protein–protein interface of the TEC and NusG with Rho in the Rho pre-termination complex is sterically incompatible with, and mutually exclusive with, the protein–protein interface of the TEC and NusG with the bacterial ribosome in the coupled transcription–translation complex^20,25,46,47^. It is attractive to hypothesize that the mechanism of quality control of transcription–translation coupling in archaea is analogous: namely, a simple steric-exclusion mechanism, based on sterically incompatible, and mutually exclusive, protein–protein interfaces for interactions of the TEC and Spt5 with FttA and interactions of the TEC and Spt5 with the archaeal ribosome. Testing this hypothesis will require the determination of a structure of the archaeal coupled transcription–translation complex and assessment of potential steric incompatibility and mutual exclusivity of the interactions therein with the interactions in the archaeal FttA pre-termination complex.

Our results define the atomic structure of the archaeal FttA pre-termination complex, suggest the likely mechanism of termination, suggest a fundamental unity in the mechanism of factor-dependent termination in bacteria, archaea, and eukaryotes, and provide a foundation for further structural and functional understanding of archaeal transcription termination and quality control in archaeal transcription–translation coupling.

## Methods

### Proteins

*Tko* FttA and FttA derivatives were prepared as described^1^, using *Escherichia coli* strain Rosetta 2 (EMD Millipore) transformed with plasmid pQE-60 (Qiagen) derivatives encoding *Tko* FttA and FttA derivatives (prepared by replacing the EcoRI-BglII segment of plasmid pQE-60 by synthetic EcoRI-BglII segments encoding *Tko* FttA and FttA derivatives; GenScript) and plasmid pMS421^50^ encoding LacI (Extended Data Fig. 1c). *Tko* RNAP, *Tko* TBP, *Tko* TFB, *Tko* Spt4, and *Tko* Spt5 were prepared as described^1^ (Extended Data Fig. 1c). Prior to sample preparation for cryo-EM structure determination, each protein sample was dialysed overnight at 4 °C against 2 litres of storage buffer (10 mM Tris-HCl, pH 7.5, 100 mM NaCl, 0.1 mM EDTA, and 5 mM dithiothreitol), concentrated by centrifugation at 4,000*g* at 4 °C through VivaSpin 10 kDa MWCO concentrators (EMD Millipore), aliquotted, flash-frozen in liquid nitrogen, and stored at −80 °C.

### Nucleic acids

Oligodeoxyribonucleotides and oligoribonucleotides (sequences in Extended Data Fig. 1a,b) were purchased from Integrated DNA Technologies, purified by PAGE, dissolved in 5 mM Tris-HCl, pH 7.5, to 0.3 mM and stored at −80 °C in aliquots.

For structural studies, nucleic acid scaffolds were prepared by mixing 60 μM nontemplate-strand oligodeoxyribonucleotide (5′-GAGTCATGATCATATTATTTTTTAGTCCAGACAGTG-3′), 55 μM template-strand oligodeoxyribonucleotide (5′-CACTGTCTGGACTGGTCGGCGCTATGATCATGACTC-3′), and 50 μM oligoribonucleotide (5′-rUrUrCrUrUrUrUrArUrCrUrUrArUrUrUrUrUrUrUrUrCrUrArUrUrUrUrUrUrCrArUrUrUrGrCrGrCrCrGrArCrC-3′ or 5′-rUrUrUrUrUrUrUrUrUrUrUrUrUrUrUrUrUrUrUrUrUrUrUrUrUrUrUrUrUrUrUrUrUrUrUrUrUrUrCrArUrUrUrGrCrGrCrCrGrArCrC-3′) in 50 μl annealing buffer (20 mM Tris-HCl, pH 7.5, and 200 mM NaCl), heating 5 min at 95 °C, and cooling to 22 °C in 1 °C steps with 30 s per step using a thermal cycler. The resulting nucleic acid scaffolds were aliquotted and stored at −80 °C.

For biochemical studies, partial nucleic acid scaffolds, comprising template-strand oligodeoxyribonucleotide and 5′-fluorescein-labelled RNA oligoribonucleotide (5′-rUrUrCrUrUrUrUrArUrCrUrUrArUrUrUrUrUrUrUrUrCrUrArUrUrUrUrUrUrCrArUrUrUrGrCrGrCrCrGrArCrC-3′) were prepared by mixing 5.5 μM template-strand oligodeoxyribonucleotide, and 5 μM fluorescein-5′-end-labelled oligoribonucleotide in 50 μl annealing buffer, heating 5 min at 95 °C, and cooling to 22 °C in 1 °C steps with 30 s per step using a thermal cycler. The resulting partial nucleic acid scaffolds were aliquotted and stored at −20 °C.

### Termination and RNA-cleavage assays

For experiments in Fig. 1a, FttA-dependent transcription termination was assessed in release assays with bead-immobilized promoter-generated TECs, detecting retained or released RNAP, as described^1^, except that *Tko* RNAP was at 40 nM, *Tko* TBP was at 80 nM, *Tko* TFB was at 80 nM, *Tko* FttA or FttA derivative was at 3 μM (monomer concentration), UTP was at 20 μM, the incubation time for transcription-initiation complex formation was 5 min at 85 °C, the incubation time for termination was 6 min at 85 °C, and RNAP subunits were detected by Coomassie brilliant blue staining.

For experiments in Fig. 1b and Extended Data Fig. 8a, FttA-dependent transcription termination and RNA cleavage were assessed in release assays with bead-immobilized promoter-generated TECs containing ^32^P-5′-end-labelled RNA, detecting retained or released RNA by storage-phosphor scanning, as described^1^, except that *Tko* RNAP was at 80 nM, *Tko* TBP was at 160 nM, *Tko* TFB was at 160 nM, *Tko* FttA or FttA derivative was at 3 μM (monomer concentration), UTP was at 1 μM, the incubation time for transcription-initiation-complex formation was 3 min at 85 °C, and the incubation time for termination and RNA cleavage was 2 min at 85 °C.

For experiments in Fig. 1c and Extended Data Fig. 8b, single-nucleotide-resolution mapping of FttA-dependent RNA cleavage was performed with TECs assembled on synthetic nucleic acid scaffolds containing a 44-nt fluorescein-5′-end-labelled RNA, detecting intact and cleaved RNA x/y fluorescence scanning, and sizing cleavage products by comparison to fluorescein-5′-end-labelled synthetic RNA markers corresponding to 22-, 24-, and 26-nt cleavage products (scaffold and marker sequences in Extended Data Fig. 1b). TECs were prepared by incubation of 0.4 μM RNAP with 0.4 μM partial nucleic acid scaffold comprising template-strand oligodeoxyribonucleotide and 5′-fluorescein-labelled RNA oligoribonucleotide in 11 μl transcription buffer (10 mM Tris-HCl, pH 8.0, 125 mM KCl, 10 mM MgCl_2_, and 1 mM dithiothreitol) for 10 min at 22 °C, followed by supplementation with 1 μl 12.5 μM nontemplate-strand oligodeoxyribonucleotide and incubation for 15 min at 22 °C. The resulting TECs were bead-immobilized by adding 2–3 μl Ni-NTA agarose beads (Thermo Fisher; pre-washed 3× 100 μl transcription buffer), incubating 15 min at 22 °C, and washing with 5× 200 μl transcription buffer. Bead-immobilized TECs were re-suspended in 12 μl transcription buffer containing 1 mM ZnCl_2_, were supplemented with 0 or 1.2 μl 30 μM Spt4/5 and incubated 2 min at 22 °C, and were supplemented with 0 or 1.8 μl 90 μM (monomer concentration) FttA or FttA derivative for Fig. 1c or with 0 or 1.8 μl 22 μM (monomer concentration) FttA or FttA derivative for Extended Data Fig. 8b, and incubated 5 min at 65 °C. Reactions were terminated by transferring reaction tubes to ice, adding 80 μl stop buffer (600 mM Tris-HCl, pH 8.0, and 30 mM EDTA) and extracting with 95 μl phenol:chloroform:isoamyl alcohol (25:24:1, v/v/v). Reaction products were precipitated by addition of 185 μl ethanol containing 15 μg GlycoBlue (Invitrogen) and centrifugation at 12,000*g* 10 min at 4 °C, were re-suspended in TBE^51^ containing 45% formamide, were heated 4 min at 99 °C, were cooled by transfer to ice, were resolved by electrophoresis on 8 M urea, 15% polyacrylamide gels (19:1 acrylami de:bis-acrylamide)^51^, were detected using x/y fluorescence scanning (Typhoon PhosphorImager, GE Healthcare), and were quantified using ImageQuant TL (GE Healthcare).

### Cryo-EM structure determination

#### Sample preparation.

Reaction mixtures containing 4 μM *Tko* RNAP, 8 μM *Tko* Spt4, 8 μM *Tko* Spt5, and 5 μM nucleic acid scaffold in 100 μl transcription buffer (20 mM HEPES-NaOH, pH 7.5, 30 mM KCl, 10 mM magnesium acetate, and 1 mM dithiothreitol) were incubated 10 min at 22 °C, and then were supplemented with 120 μl 15 μM FttA(H255A/H591A) (monomer concentration) and incubated 10 min at 22 °C and 5 min at 42 °C. Reaction mixtures were concentrated to 35 μl by centrifugation 12 min at 20,000*g* at 4 °C through pre-chilled Amicon Ultra-0.5 30 KDa MWCO concentrators (EMD Millipore), were supplemented with 4 μl of ice-cold 80 mM CHAPSO (Hampton Research), and were kept on ice until applied to electron microscopy grids.

Electron microscopy grids were prepared using a Vitrobot Mark IV autoplunger (FEI/Thermo Fisher), with the environmental chamber set to 22 °C and 100% relative humidity. Samples (3 μl) were applied to Quantifoil UltrAuFoil 1.2/1.3 grids (Quantifoil) glow-discharged 300 s using a PELCO glow-discharge system (Ted Pella); grids then were blotted with no. 595 filter paper (Ted Pella) for 1 s, flash-frozen by plunging in liquid ethane cooled with liquid N_2_, clipped, and stored in liquid N_2_.

#### Data collection and data reduction for *Tko* FttA(H255A/H591A)–Spt4–Spt5–TEC^44^.

Cryo-EM data were collected at Brookhaven National Laboratory (BNL) Laboratory for BioMolecular Structure, using a 300 kV Krios Titan electron microscope (FEI/Thermo Fisher) equipped with a Gatan K3 Summit direct electron detector (Gatan). Movies were collected in super-resolution mode using EPU (Thermo Fisher) at a nominal magnification of 105,000×, a calibrated pixel size of 0.825 Å per pixel, and a dose rate of 22 e− Å^−2^ s^−1^. Movies were recorded at 56.5 ms per frame for 2.26 s (40 frames), resulting in a total radiation dose of 50 e^−^ Å^−2^. The defocus range varied from −1.0 μm and −2.0 μm. A total of 9,398 movies were recorded from one grid over 2 days.

Data were processed as summarized in Extended Data Fig. 2a. Data processing was performed using cryoSPARC v4.2.1^52^. Following patch motion correction, patch contrast transfer function (CTF) estimation, and manual inspection to remove micrographs with ice contamination or poor image quality, particles were picked from a random subset of 964 micrographs using cryoSPARC Blob Picker, 2D classification was performed to generate a 2D template, and particles were autopicked from 8,687 micrographs using the 2D template and cryoSPARC Template Picker, yielding a total of 1,292,792 particles. Particles were extracted into 200 × 200 pixel boxes (2 × 2 downscaled) and subjected to 2 rounds of reference-free 2D classification and removal of poorly populated 2D classes, yielding a selected set of 917,011 particles. The selected set was used for ab initio reconstruction (*n* = 6) to generate an initial 3D model, and was subjected to 2 rounds of heterogeneous refinement (*n* = 6) to remove poorly populated 3D classes, yielding a selected class of 289,780 particles. The selected class was re-extracted into 400 × 400 pixel boxes and subjected to non-uniform refinement (*n* = 4), yielding a selected subclass, containing 171,269 particles, exhibiting unambiguous density for FttA, the RNAP main mass (RpoA′, RpoA″, RpoB, RpoD, RpoH, RpoK, RpoL, RpoN, and RpoP), the RNAP stalk (RpoE and RpoF), and Spt5 KOW, and exhibiting high particle homogeneity. (Other subclasses lacked density for the RNAP stalk and/or exhibited low particle homogeneity.) The selected subclass was subjected to non-uniform refinement with global CTF refinement, yielding a reconstruction with global resolution of 2.5 Å as determined from gold-standard Fourier shell correlation (map 1a; Extended Data Fig. 2a,d,f-h). To improve map quality for FttA, RNAP stalk, and Spt5 KOW, the selected subclass was subjected to local refinement using a mask for FttA, RNAP stalk, and Spt5 KOW, yielding a locally refined map for FttA, RNAP stalk, and Spt5 KOW with a resolution of 2.7 Å as determined from gold-standard Fourier shell correlation (map 1b; Extended Data Fig. 2a,e,i-k). The global map (map 1a) and the locally refined map (map 1b) were combined in Chimera 1.16^53^ using the ‘vop maximum’ command to generate a composite map for model building and model refinement. The reconstruction showed clear density for two FttA protomers (FttA^prox^ and FttA^dist^), Spt4, Spt5, and the TEC (Extended Data Fig. 3a-j).

The initial atomic model for the FttA pre-termination complex complex (*Tko* FttA(H255A/H591A)–Spt4–Spt5–TEC*^44^*) was built by manual docking in Chimera 1.16^53^ of: (1) a cryo-EM structure of *Tko* RNAP (PDB 6KF9)^54^; (2) a homology model of *Tko* FttA built based on a crystal structure of *Pyrococcus horikoshii* FttA (PDB 3AF5)^5^; and (3) homology models of *Tko* Spt4, *Tko* Spt5 NGN, and *Tko* Spt5 KOW built based on a crystal structure of a *Pyrococcus furiosus* Spt4–Spt5 complex (PDB 3P8B)^55^. DNA and RNA were manually built using Coot^56^. For the FttA^prox^ N terminus (residues 1–3), Spt4 N terminus (residues 1–3), Spt5 N terminus (residues 1–3), RNAP RpoA′ N terminus (residues 1–3), RNAP RpoA″ N terminus (residue 1), RNAP RpoB N terminus and flap tip (residues 1–8 and 822–833), RNAP RpoC N terminus (residues 1 and 83–91), RpoD N terminus (residue 1), RpoH N terminus (residues 1–5), RpoK N terminus (residue 1), and RpoP N terminus (residues 1–5), density was absent, suggesting high segmental flexibility; these segments were not fitted.

The initial atomic model was subjected to real-space rigid-body refinement in Phenix^57^, and was subjected to iterative cyles of model building in Coot^56^ and refinement in Phenix^57^. Structure visualization was performed using PyMOL (Schrödinger).

The final density maps and atomic coordinates were deposited in the Electron Microscopy Data Bank with accession codes EMD-44438 (composite map), EMD-44454 (global map), and EMD-44649 (local map processed by focussed refinement for FttA, RNAP stalk, and Spt5 KOW) and in the Protein Data Bank with accession code PDB 9BCT.

#### Data collection and data reduction for *Tko* FttA(H255A/H591A)–Spt4–Spt5–TEC^52^.

Cryo-EM data were collected at the National Center for CryoEM Access and Training (NCCAT), using a 300 kV Krios Titan electron microscope (FEI/Thermo Fisher) equipped with a Falcon4EC (Thermo Fisher) imaging system. Movies were collected using Leginon^58^ at a nominal magnification of 165,000×, a calibrated pixel size of 0.7304 Å per pixel, and a dose rate of 8 e^−^ Å^−2^ s^−1^. Movies were recorded at 50 ms per frame for 6 s (120 frames), resulting in a total radiation dose of 48 e^−^ Å^−2^. The defocus range varied from 0.6 μm and −2.0 μm. A total of 16,441 movies were recorded from two grids over 3 days.

Data were processed as summarized in Extended Data Fig. 4a, using procedures as in the preceding section. Data processing yielded reconstructions at 2.2 Å resolution (overall map; map 2a) and 2.3 Å resolution (local map, processed by focussed refinement for FttA, RNAP stalk, and Spt5 KOW; map 2b) (Extended Data Fig. 4a,e,i-k and Extended Data Table 1). Atomic models were built and refined as in the preceding section.

The final density maps and atomic coordinates were deposited in the Electron Microscopy Data Bank with accession codes EMD-44439 (composite map), EMD-44455 (global map), and EMD-44650 (local map processed by focussed refinement for FttA, RNAP stalk, and Spt5 KOW) and in the Protein Data Bank with accession code PDB 9BCU.

## Extended Data

**Extended Data Table 1 ∣:** Cryo-EM data collection, refinement, and validation statistics

	FttA^H255A;H591A^-Spt4-Spt5-TEC^44^ (EMDB-44438; EMD-44454; EMD-44649; PDB 9BCT)	FttA^H255A;H591A^-Spt4-Spt5-TEC^52^ (EMDB-44439; EMD-44455; EMD-44650; PDB 9BCU)
Data collection and processing		
Magnification	105,000	165,000
Voltage (kV)	300	300
Electron exposure (e^−^/Å^2^)	50	48.33
Defocus range (μm)	−1.0 to −2.2	−0.6 to −2.0
Pixel size (Å)	0.825	0.73
Symmetry imposed	C1	C1
Initial particle images (number)	1,292,792	977,159
Final particle images (number)	171,269	137,835
Map resolution (Å)	2.5	2.2
FSC threshold	0.143	0.143
Map resolution range (Å)	2-5	2-5
Refinement		
Initial model used (PDB code)	6KF9, 3AF5, 3P8B	9BCT
Model resolution (Å)	2.1	1.8
FSC threshold	0.143	0.143
Model resolution range (Å)	2-5	2-5
Map sharpening *B* factor (Å^2^)	−56.6	−40.7
Model composition		
Non-hydrogen atoms	39,772	39,765
Protein residues	4,785	4,785
Ligands	8, 1	8, 1
*B* factors (Å^2^)		
Protein	80.04	65.77
Ligand	121.11	105.63
R.m.s. deviations		
Bond lengths (Å)	0.002	0.002
Bond angles (°)	0.532	0.446
Validation		
MolProbity score	1.70	1.52
Clash score	7.12	4.59
Poor rotamers (%)	2.15	2.37
Ramachandran plot		
Favored (%)	97.75	98.02
Allowed (%)	2.25	1.98
Disallowed (%)	0.00	0.00

## Supplementary Material

Supplemental Materials

## Data Availability

Cryo-EM maps and atomic coordinates generated in this work are available from the Electron Microscopy Database (EMDB accession codes EMD-44438, EMD-44454, EMD-44439, EMD-44455, EMD-44649, and EMD-44650) and the Protein Database (PDB accessions 9BCT and 9BCU). Unique biological materials will be made available to qualified investigators on request.
