# Supplementary figures and images for: Structural basis of archaeal FttA-dependent transcription termination
Source: Nature. Author manuscript; Available in PMC 2025 May 1. (PMC11616081; doi:10.1038/s41586-024-07979-9)

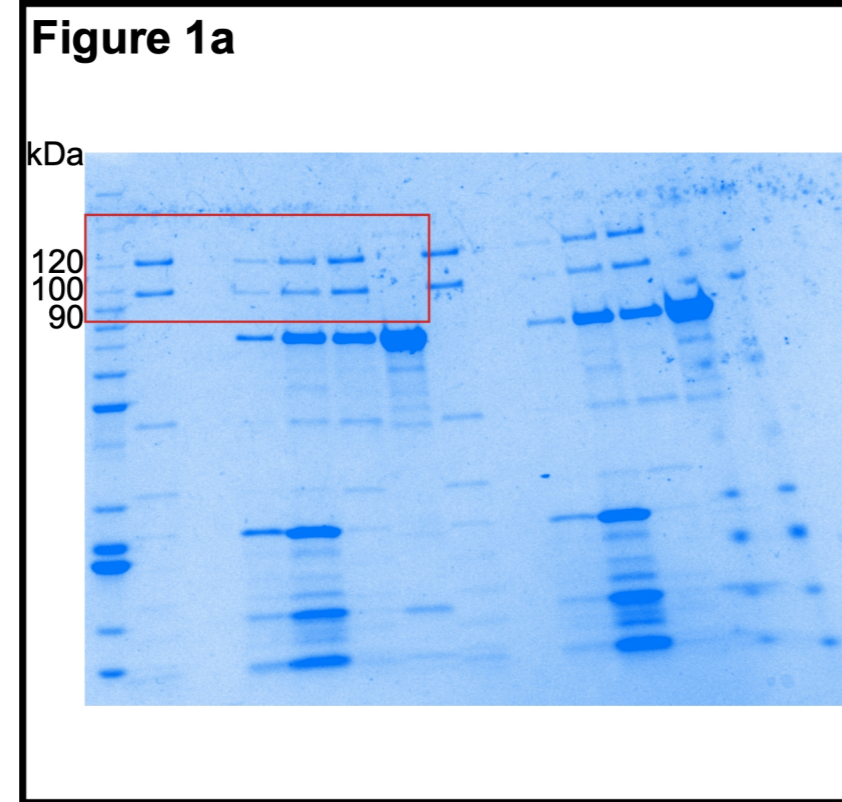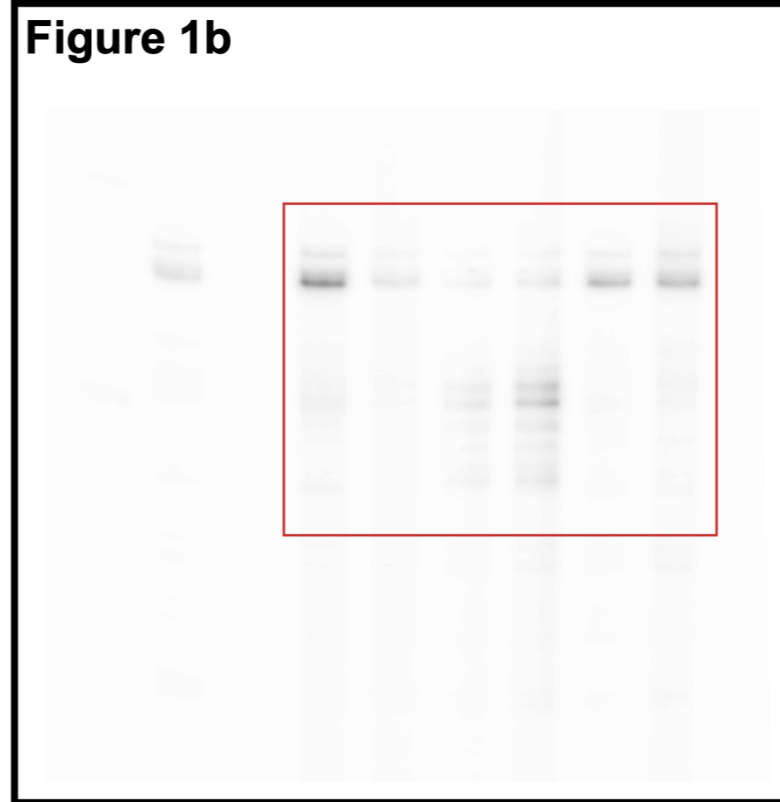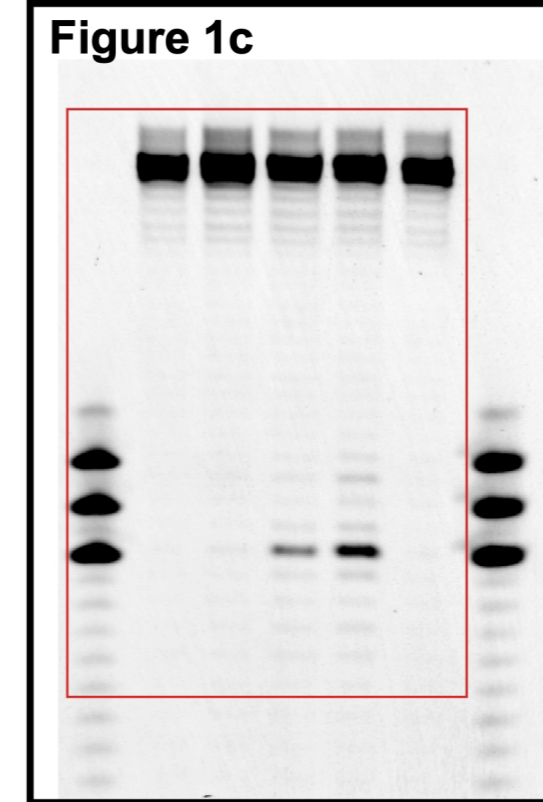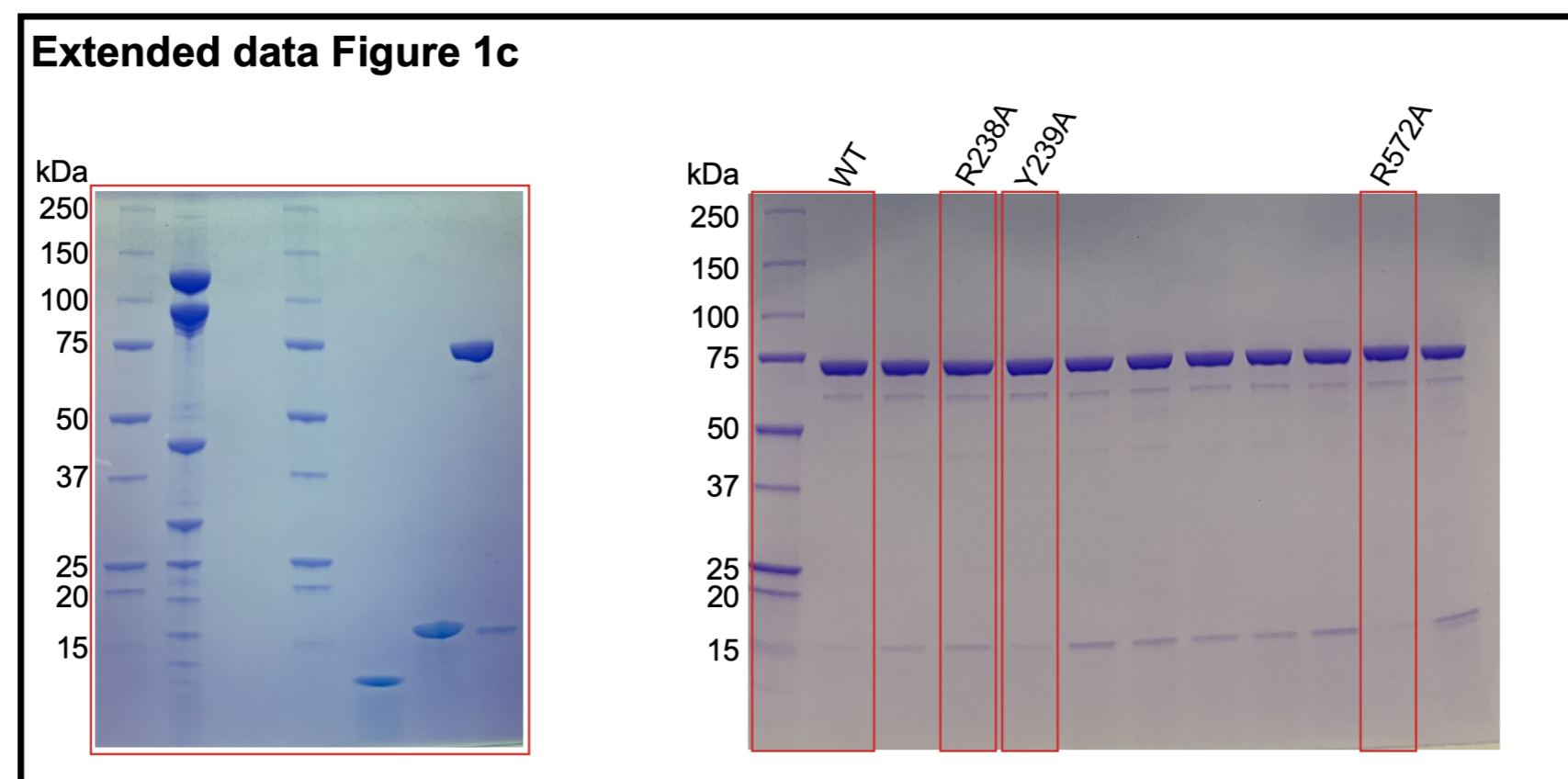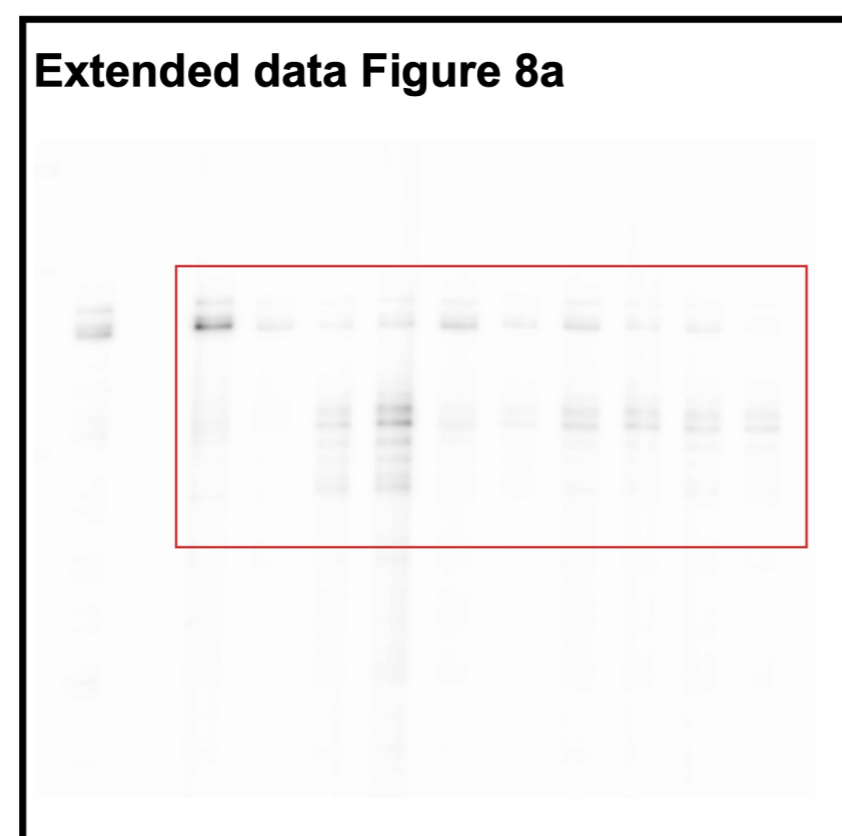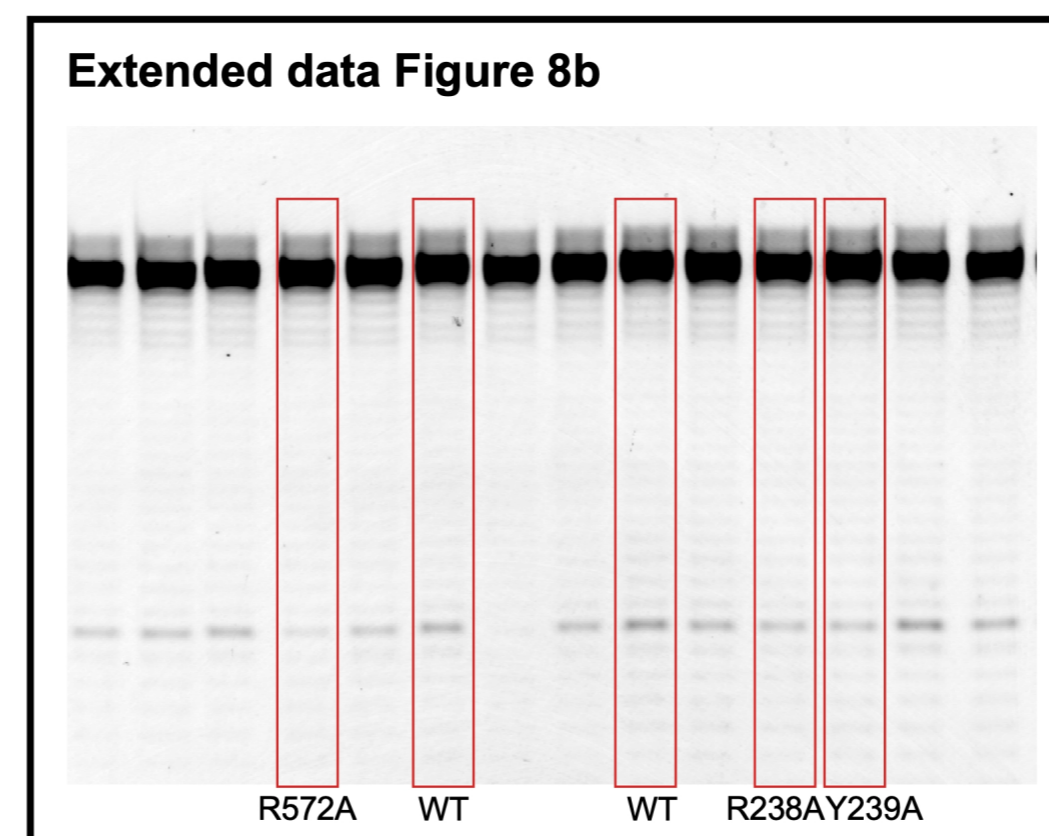

Supplement: Supplemental Materials [file NIHMS2036802-supplement-Supplemental_Materials.pdf]
